# The Continuum Approach to the Description of Semi-Crystalline Polymers Deformation Regimes: The Role of Dynamic and Translational Defects

**DOI:** 10.3390/polym10101155

**Published:** 2018-10-16

**Authors:** Yurii V. Grinyaev, Nadezhda V. Chertova, Evgeny V. Shilko, Sergey G. Psakhie

**Affiliations:** 1 Institute of Strength Physics and Materials Science SB RAS, pr. Akademicheskii 2/4, 634055 Tomsk, Russia; chertova@ispms.ru (N.V.C.); shilko@ispms.tsc.ru (E.V.S.); sp@ispms.ru (S.G.P.); 2 Physical Faculty, National Research Tomsk State University, 36 Lenin ave., 634050 Tomsk, Russia

**Keywords:** semi-crystalline polymers, two-phase model, continuum defects theory, elasticity, plasticity, viscosity, translational defects, dynamic defects

## Abstract

This paper presents a new approach to describe the mechanical behavior of semi-crystalline polymers, the plastic deformation of which is determined by their two-phase structure. To describe the plastic behavior of semi-crystalline polymers, a two-phase model is used. In the framework of this model, one phase is in a hard (crystalline) state, and the other in a soft (amorphous) state. The two-phase material is modeled by a single-phase homogeneous continuum based on the approximation of the effective medium. It is assumed that two infinitely close material points of the continuum are connected in series by elastic and viscous bonds, which corresponds to the Maxwell model. It is shown that, in this case, the Maxwell continuum is a pseudo-Euclidean space. Generalizing the definition of defects from a three-dimensional space to a four-dimensional pseudo-Euclidean space, we obtained a dynamic system of nonlinear, interrelated equations to describe the behavior of translational-type defects in the solid phase and dynamic defects in the amorphous phase. As an example of an application for these equations, the phenomenon of creep under uniaxial loading is considered. It is shown that the formalism of the proposed two-phase model makes it possible to describe creep phenomenon regularities, which correspond to both the aging theory and the flow theory.

## 1. Introduction

Many materials have neither the long-range order of ideal crystalline bodies nor the completely random structure of ideal liquids. In recent decades, such materials became the object of intensive experimental and theoretical research, as determined by their wide practical application. Materials that simultaneously possess the properties of an elastic body and liquid are called “soft matter” [1,2]. Duality in the behavior of “soft” materials determines the nonlinearity of their response and the delay in the reaction to mechanical loading, even in the region of reversible strains (macroscopically, such behavior is traditionally described using viscoelastic rheological models). In addition to viscoelastic properties, some “soft materials” exhibit the property of plasticity [3]. These include, in particular, semi-crystalline polymers.

It is well known that, at a basic microscopic level, semi-crystalline polymers consist of ribbon-like lamellar crystals separated from each other by amorphous layers, and all of them are held by molecular chains crossing the interface between the crystal and amorphous phases [4,5,6]. As a result of such cohesion, the crystalline lamellae and amorphous layers are deformed consistently to maintain the continuity of the material [7,8]. Crystalline lamellae are deformed as elastic solids. Depending on temperature the amorphous interlayers can be deformed as glassy, highly elastic, or viscous materials.

To describe the plastic behavior of semi-crystalline polymers, a two-phase model was proposed in Reference [9]. This model was generalized in References [10,11]. According to the proposed model, the semi-crystalline polymer represents a two-phase system consisting of a solid phase (crystalline lamellae and a bonded amorphous layer at the interface of a crystalline lamella) and a soft phase (a free amorphous volume). Thus, Reference [11] considers an analogy between semi-crystalline polymers and porous materials, filled with fluids. An analogy is drawn between the fluid filling the pore space and the free amorphous phase filling the intercrystalline space in the polymer. Such an analogy is most complete if one assumes that the amorphous phase is in the viscous flow state. Note that two-phase models that consider the cooperative contribution of crystalline and amorphous phases in the mechanical behavior of semi-crystalline polymers became popular in recent years [12,13,14,15].

It was established in References [16,17,18,19,20,21,22,23,24,25,26,27,28,29,30,31] that the crystalline polymer phase is deformed according to classical crystallographic mechanisms—nucleation and a subsequent slip of dislocations. Thus, Reference [16] presents the results of an analysis of the deformation response of solids. These solids have different nature and structure: crystalline, glassy, organic, inorganic, and low- and high-molecular. The presented experimental data show the generality of the mechanical behavior of various solid materials, including the presence of a yield point, as well as localized shear bands, which are a consequence of dislocations sliding in metallic materials. References [18,19,20,21,23,24,25,26,28,29,30,31] report the results of the study of shear transformation zones in polymers. Shear transformation zones are places of localized plastic strains possessing significant elastic stresses, analogous to stress fields from dislocations. Investigations carried out in References [11,16,29] showed that active crystallographic processes occur in polymers at moderate strains, like in metals, with nucleation of local shears in the form of a relative slip of weakly bonded long polymeric molecules. Microscale strain localization in the form of local shears can lead to macroscopic localization in the form of necking [32], shear banding [33], and others.

The results of experimental studies given in Reference [22] convincingly indicate that, in the range of moderate strains, polymers are plastically deformed according to the classical mechanisms of nucleation of local shear zones. In particular, the displacement fields around a dislocation core were calculated in Reference [34]. Therefore, a large group of polymer plastic deformation theories is based on the mathematical apparatus and postulates of the general theory of crystalline bodies. It was proposed to use the concept of dislocation to describe polymer plasticity. In this case, the relationships from the theories of plasticity of crystalline materials, the Orovan ratio, the scalar density of defects, and so on are applied [18]. In particular, several studies consider macroscopic phenomenological theories of the viscoelastic–plastic behavior of polymers. They are based on the multiplicative or additive decomposition of total distortion of the material elementary volume into elastic and plastic components [29,30]. However, these papers do not discuss the nature of these contributions and do not take into account that both elastic and plastic strains can, in turn, be decomposed into components. It is well known that defects provide elastic and plastic distortions in the material, which do not meet the compatibility condition separately. During the process of deformation, the effective fields of elastic strains are formed in the material due to external loading and internal defects. Elastic strains from external loading (compatible strains) vanish when external loading is removed, whereas elastic incompatible strains from defects remain in the material and create internal stress fields. To describe these internal stresses, the introduction of internal parameters is needed [31]. Complete plastic strains consist of incompatible plastic strains from defects and compatible (irreversible) plastic strains from defects that disappear during the process of deformation. Note that irreversible (compatible) strains do not cause internal stresses and characterize the energy dissipated during deformation.

It is important to note that, in the above theoretical studies, the amorphous phase has a small role, namely the role of the stress transmitter between the crystallites. However, due to the interaction between the crystalline and amorphous layers, which must be deformed in accordance with the continuity of the material, the amorphous component must take on a more important and even key role. At present, there are actually no macroscopic models that include equations describing the dynamics and interrelationships of translational defects in the crystalline phase and defects in the amorphous (soft) phase. The aim of this paper was to construct a closed system of interrelated dynamic equations for defects arising during deformation in the crystalline and amorphous phases within the framework of the continuum defects theory [35]. In order to build this system, we used the basic principles of the two-phase model proposed in Reference [9,10,11] and the analogy with a porous material filled with fluid. In the present work, translational defects in the crystalline phase were characterized using the density tensor and the defect flux density tensor, and defects in the amorphous phase were described by vector characteristics.

## 2. Materials and Methods

### 2.1. Definition of a Semi-Crystalline Polymer as a Two-Phase Material

We consider a semi-crystalline polymer as a two-phase material consisting of crystalline and amorphous phases. We make an analogy between a fluid filling the pore space and a free amorphous phase filling the intercrystalline space in the polymer [9,10,11].

It is assumed that the representative volume of the polymer contains both components. Densities, volume fractions, elastic and viscous characteristics, and other parameters are determined for each component in this volume. In the approximation of an effective medium, it is possible to estimate the effective characteristics of a representative volume as a whole. The density is defined as

(1) $$\rho =n_{1}\rho_{1}+n_{2}\rho_{2},\;n_{1}+n_{2}=1,$$

where *n_k_* and ρ*_k_* (*k* = 1, 2) are the volume fractions of the phases (components) in a representative volume and densities of the phases, respectively. Hereafter, we assume that the value of the subscript *k* = 1 refers to the amorphous component of the semi-crystalline polymer, and *k* = 2 corresponds to the crystalline component. The effective elastic characteristics and the dynamic viscosity of a representative volume are determined using Voight averaging,

(2) $$K=n_{1}K_{1}+n_{2}K_{2},\;\eta =n_{1}\eta_{1}+n_{2}\eta_{2},$$

or Reuss averaging,

(3) $$K=\frac{K_{1}K_{2}}{n_{1}K_{2}+n_{2}K_{1}},\;\eta =\frac{\eta_{1}\eta_{2}}{n_{1}\eta_{2}+n_{2}\eta_{1}},$$

where *K_k_* are the elastic constants (the shear or bulk modulus) of the phases, and η*_k_* are the dynamic viscosities of the phases. We note that the Voight averaging is regarded as an upper bound, and the Reuss averaging as an estimate from below. Sometimes the average arithmetic mean or average geometric mean obtained with Voight and Reuss averaging is used. Under the assumptions made above, we pass from a two-component medium to a one-component effective medium at each material point, from which mechanical parameters are determined. Furthermore, we assume that two infinitely close material points of the effective medium are connected in series through elastic and viscous bonds, which correspond to the Maxwell continuum. Note that a multi-element model with parallel-connected Maxwell arms was applied in References [36,37] to describe hardening, relaxation processes, and so on. In our case, these processes are described by means of considering the defects arising in crystalline and amorphous components.

When connecting material points through elastic and viscous elements, the total value of the infinitesimal increment of their relative displacement $d\vec{u}$ is composed of elastic ($d{\vec{u}}^{el}$) and viscous ($d{\vec{u}}^{lq}$) displacements:

(4) $$d\vec{u}=d{\vec{u}}^{el}+d{\vec{u}}^{lq}.$$

Elastic displacements are used to describe the deformation of a crystalline component of the polymer, and viscous displacements are used for the amorphous component.

Viscous displacements are described using hydrodynamic parameters:

(5) $$d{\vec{u}}^{lq}=\vec{V}dt,$$

where $\vec{V}$ is the local velocity of the “fluid” flow, and *t* is the time. Hereafter, the term “fluid” is conventional and it characterizes the ability of the phase to viscous flow. The flow velocity can be expressed in terms of the potential. Let us suppose that the viscous phase is filtered in a porous medium representing the crystalline phase. If gravitational forces are not taken into account, then Darcy’s law can be applied as the linear approximation in the case of the laminar flow of fluid:

(6) $${\vec{V}}^{\prime }=-(k/\mu )\nabla P,$$

where ${\vec{V}}^{\prime }$ is the filtration velocity, *k* is the permeability of the porous medium, μ is the viscosity coefficient of the fluid, and *P* is the pressure in the fluid. The filtration velocity does not coincide with the velocity of the fluid particles, and is related as follows:

(7) $$\vec{V}={\vec{V}}^{\prime }/\lambda ,$$

where λ is the porosity. If the porosity is equal to one, then we come to free fluid flow. In this case, Darcy’s law can be presented as

(8) $$\vec{V}=-(k/\mu )\nabla P=-\nabla \Phi ,$$

where *k* and μ are both constants, and Φ is a potential. It follows that the motion of the fluid is directed from areas with a large potential to areas with a lower potential, which leads to equalization of the potential and equilibrium.

The change in the distance between two infinitely close material points in the case of small strains is defined as

(9) $$\begin{array}{l} ds^{2}-ds_{0}^{2}=d\vec{u}\cdot d\vec{r}=(d{\vec{u}}^{el}+d{\vec{u}}^{lq})\cdot d\vec{r}=(d{\vec{u}}^{el}+\vec{V}dt)\cdot d\vec{r}=\\ =(d{\vec{u}}^{el}-\nabla \Phi dt)\cdot d\vec{r}=d{\vec{u}}^{el}\cdot d\vec{r}-d\Phi dt=d{\vec{u}}^{el}\cdot d\vec{r}-\frac{d\Phi }{c}cdt \end{array},$$

where $ds_{0}$ and ds are the distances between infinitely close material points at times *t* and *t + dt*, $d\vec{r}=\left(dx,dy,dz\right)$ is a three-dimensional (3D) vector connecting two infinitely close material points at the initial moment of time, and *c* is an unknown constant, which has a dimensionality of velocity. The expression $d{\vec{u}}^{el}\cdot d\vec{r}-\left(d\Phi /c\right)cdt$ is a scalar product of two four-dimensional (4D) vectors: the four-dimensional relative displacement vector $d\vec{U}=\left(du_{1}^{el},du_{2}^{el},du_{3}^{el},d\Phi /c\right)$ and the four-dimensional radius vector $d\vec{R}=\left(dx,dy,dz,cdt\right)$ with contravariant coordinates in the pseudo-Euclidean space. This expression can be written as follows:

(10) $$d\vec{u}\cdot d\vec{r}-\frac{d\Phi }{c}cdt=dU^{\tau }dR^{\gamma }q_{\tau \gamma },$$

where

(11) $$q^{\tau \gamma }=q_{\tau \gamma }=\{\begin{array}{l} -1,\;\tau =\gamma =0,\;time\;component\\ 1,\;\tau =\gamma =1,2,3,\;spatial\;components\\ 0,\;\tau \neq \gamma \end{array}$$

is the metric tensor of the pseudo-Euclidean space with the help of which it is possible to raise and lower indices:

(12) $$dU_{\tau }=dU^{\gamma }q_{\gamma \tau },\;dU^{\gamma }=dU_{\tau }q^{\tau \gamma }.$$

Hereafter, Greek indices τ, γ, η… run through the values 0, 1, 2, 3, and Latin indices *i*, *j*, *n*, *m*… accept only spatial indices 1, 2, 3. Thus, the considered two-phase liquid-crystal medium within the linear approximation is a pseudo-Euclidean space in which the relative displacements vector $d\vec{U}$, along with the spatial components $du_{1}^{el},du_{2}^{el},du_{3}^{el}$, acquires a time component dΦ/c, and time becomes an equitable coordinate along with spatial coordinates. In this case, we can introduce a four-dimensional displacement vector:

(13) $$U^{\tau }=\left(\frac{\Phi }{c},u_{1}^{el},\;u_{2}^{el},\;u_{3}^{el}\right)\;\mathrm{and}\;U_{\tau }=\left(-\frac{\Phi }{c},u_{1}^{el},\;u_{2}^{el},\;u_{3}^{el}\right).$$

The vector has three spatial components $\left(u_{1}^{el},\;u_{2}^{el},\;u_{3}^{el}\right)$ from the crystalline phase and one time component Φ/c from the amorphous phase.

### 2.2. Macroscopic Definition of Defects in the Classical 3D Continuum Theory of Crystalline Solids

The model developed in this paper is based on the principles of the classical three-dimensional continuum theory of crystalline solids with defects [35] and generalizes these principles. Within the framework of the classical theory, the main deformation characteristic of a material is the three-dimensional tensor of distortion (3-tensor of distortion) $\beta_{ij}$. It consists of two contributions: (1) distortion $\beta_{ij}^{ext}$ due to external loading, and (2) distortion $\beta_{ij}^{\mathrm{int}}$ caused by the presence of defects [38,39]:

(14) $$\beta_{ij}=\beta_{ij}^{ext}+\beta_{ij}^{\mathrm{int}}=\beta_{ij}^{ext}+\beta_{ij}^{\mathrm{int},el}+\beta_{ij}^{\mathrm{int},pl}.$$

Distortion $\beta_{ij}^{ext}$ is reversible (it becomes zero when removing external loads) and expressed through the gradients of elastic displacements (distortions in the elastic deformation of the material). It is related to external stresses σ*_ij_* by means of a given material relationship (Hooke’s law). Defects create the fields of distortion $\beta_{ij}^{\mathrm{int}}$ in the material. These fields include two components: elastic distortions $\beta_{ij}^{\mathrm{int},el}$ (and associated elastic stress fields) and plastic distortions $\beta_{ij}^{\mathrm{int},pl}$. We note that the relationship between $\beta_{ij}^{\mathrm{int},el}$ and the field of elastic stresses from defects is determined using Hooke’s law. Assuming that the defects do not violate the continuity of the material, the distortion $\beta_{ij}^{\mathrm{int}}$ is a complete differential:

(15) $$\beta_{ij}^{\mathrm{int}}=\beta_{ij}^{\mathrm{int},el}+\beta_{ij}^{\mathrm{int},pl}=\nabla \vec{\upsilon },$$

where $\vec{\upsilon }$ is some displacement vector (caused by defects), which cannot be represented as a sum of elastic and plastic displacement vectors. This relationship has the following implication: when defects disappear during deformation (this can be a result of defect escape to the surface, annihilation, etc.), the elastic distortions turn to zero ($\beta_{ij}^{\mathrm{int},el}$ = 0), but plastic distortions $\beta_{ij}^{\mathrm{int},pl}$ retain their value. In this case, $\beta_{ij}^{\mathrm{int},pl}$ become compatible distortions, that is, they are expressed in terms of the displacement gradient (the displacements themselves can be treated as plastic displacements in this case) and characterize the irreversible forming of the material. Accounting for elastic and plastic distortions caused by defects is a key statement of the considered theory, which distinguishes it from the classical models of plasticity of solids.

Macroscopic continuum theory assumes description of defects using the defect density tensor. This tensor is defined as $\nabla \times \beta_{ij}^{\mathrm{int},pl}$. Using the continuity relationship (Equation (15)), one can obtain the formulation of the components of this tensor through the spatial derivatives of the tensor of elastic distortion from defects $\beta_{ij}^{\mathrm{int},el}$:

(16) $$\alpha_{ijk}=\frac{\partial \beta_{jk}^{\mathrm{int},el}}{\partial x^{i}}-\frac{\partial \beta_{ik}^{\mathrm{int},el}}{\partial x^{j}}.$$

Note that defect density tensor characterizes the density of translational defects.

Dynamics of the inelastic deformation of a crystalline solid is determined by the dynamics of redistribution (motion) of defects. Within the continuum theory, these dynamics are integrally (macroscopically) characterized using the tensor of the density of defect flux *J_ji_* through the outer contour that limits the considered volume of material [38,39]:

(17) $$J_{ji}=\frac{\partial \beta_{ji}^{\mathrm{int},el}}{\partial t}-\frac{\partial \nu_{i}}{\partial x^{j}}=-\frac{\partial \beta_{ji}^{\mathrm{int},pl}}{\partial t},$$

where $\vec{\nu }=\left(\nu_{1},\nu_{2},\nu_{3}\right)$ is the velocity of displacement caused by defect fluxes. Note that the vector $\vec{\nu }$ is not a time-derivative of the vector of elastic displacement ${\vec{u}}^{el}$. The above-shown definition of the defect flux density is justified by the fact that, in the case of compatible elastic strain, when the vector of elastic displacements is defined (defect-free material), the defect density tensor and the defect flux density tensor must automatically vanish. One can see that this follows from the definition of *J_ji_*.

The system of governing equations of the classical three-dimensional theory includes the equations of defect field dynamics (including equations for the defect density tensor and defect flux density tensor), as well as the constitutive (material) equations connecting local stresses caused by external load and internal defects with $\beta_{ij}^{ext}$ and $\beta_{ij}^{\mathrm{int},el}$ correspondingly (they are formulated on the basis of Hooke’s law). Note that dynamic equations take into account the interaction between translational defects.

### 2.3. Definition of Defects in the Developed 4D Continuum Model of Semi-Crystalline Polymers

The presence of an amorphous (“soft”) phase in the bulk of the material, in addition to the crystalline phase, leads to the need to generalize not only the displacement vector, but also the distortion tensor and the defect density tensor. In this case, they all become four-dimensional and include the dynamic components associated with the defect fluxes in the crystalline component and the dynamic defects in the “fluid-like” amorphous component.

In particular, the 4D tensor (4-tensor) of distortion consists of two contributions:

(18) $$\beta_{\tau \gamma }=\beta_{\tau \gamma }^{ext}+\beta_{\tau \gamma }^{\mathrm{int}}.$$

The first contribution corresponds to the strain of a defect-free polymer, in which the crystalline component is elastically deformed, and the amorphous component flows in the laminar regime. This contribution is defined as a 4D gradient (4-gradient) of the 4D displacement (4-displacement) vector (13):

(19) $$\beta_{\tau \gamma }^{ext}=\nabla^{\tau }U_{\gamma }=\left|\begin{array}{cccc} -\frac{1}{c^{2}}\frac{\partial \Phi }{\partial t} & \frac{1}{c}\frac{\partial u_{1}^{el}}{\partial t} & \frac{1}{c}\frac{\partial u_{2}^{el}}{\partial t} & \frac{1}{c}\frac{\partial u_{3}^{el}}{\partial t}\\ -\frac{1}{c}\frac{\partial \Phi }{\partial x_{1}} & \frac{\partial u_{1}^{el}}{\partial x_{1}} & \frac{\partial u_{2}^{el}}{\partial x_{1}} & \frac{\partial u_{3}^{el}}{\partial x_{1}}\\ -\frac{1}{c}\frac{\partial \Phi }{\partial x_{2}} & \frac{\partial u_{1}^{el}}{\partial x_{2}} & \frac{\partial u_{2}^{el}}{x_{2}} & \frac{\partial u_{3}^{el}}{\partial x_{2}}\\ -\frac{1}{c}\frac{\partial \Phi }{\partial x_{3}} & \frac{\partial u_{1}^{el}}{\partial x_{3}} & \frac{\partial u_{2}^{el}}{\partial x_{3}} & \frac{\partial u_{3}^{el}}{\partial x_{3}} \end{array}\right|,\;\tau ,\gamma =0,1,2,3,$$

where the del operator has the following components: $\nabla^{\tau }=\frac{\partial }{\partial x^{\tau }}=\left(\frac{\partial }{c\partial t},\;\frac{\partial }{\partial x_{1}},\;\frac{\partial }{\partial x_{2}},\;\frac{\partial }{\partial x_{3}}\right)$ and $\nabla_{\tau }=\frac{\partial }{\partial x_{\tau }}=\left(-\frac{\partial }{c\partial t},\;\frac{\partial }{\partial x_{1}},\;\frac{\partial }{\partial x_{2}},\;\frac{\partial }{\partial x_{3}}\right)$. The spatial components of this tensor ($\beta_{ij}^{ext}$) are the local result of the action of external (applied) load and are related to the corresponding local stresses through a material relationship (Hooke’s law). The components of the tensor with zero value of one of the indices (τ or γ) are related to elastic strain rates of the crystalline phase or rates of laminar flow of the amorphous phase. These components are associated with the dissipation of the kinetic energy of the phases. The rate of dissipation is determined on the basis of a given law of viscous friction.

The presence of defects of the translational type in the crystalline component causes the elastic $\beta_{ij}^{el}$ and plastic distortions $\beta_{ij}^{pl}$ [38,39], in addition to the distortion (Equation (19)) of the defect-free polymer. We note that, in a material with defects, the vector of displacements associated with these defects is not defined. Therefore, the distortions $\beta_{ij}^{el}$ cannot be defined as the gradient of the field of elastic displacements and are considered as independent deformation parameters. In addition, defect fluxes in the crystalline phase cause the motion rates $\vec{\nu }=\left(\nu_{1},\nu_{2},\nu_{3}\right)$, which cannot be defined as the time-derivative of displacements and are also considered as separate (independent) parameters of the model. By analogy, if the flow of the amorphous (viscous) component is non-laminar (non-potential), the velocity vector of non-laminar flow $\vec{V}=\left(V_{1},V_{2},V_{3}\right)$ is not defined as a gradient of some potential and is a separate parameter. In such a consideration, the primary characteristic of the effect of defects on the deformed state of the effective medium is the 4-tensor of distortion of the following form:

(20) $$\beta_{\tau \gamma }^{\mathrm{int}}=\left|\begin{array}{cccc} \frac{1}{c^{2}}\Psi & \frac{1}{c}v_{1} & \frac{1}{c}v_{2} & \frac{1}{c}v_{3}\\ \frac{1}{c}V_{1} & \beta_{11}^{el} & \beta_{12}^{el} & \beta_{13}^{el}\\ \frac{1}{c}V_{2} & \beta_{21}^{el} & \beta_{22}^{el} & \beta_{23}^{el}\\ \frac{1}{c}V_{3} & \beta_{31}^{el} & \beta_{32}^{el} & \beta_{33}^{el} \end{array}\right|.$$

Note that the potential Ψ, which determines the component ($\beta_{00}^{\mathrm{int}}$), is not defined in terms of the potential Φ in Equation (19), and is also an independent parameter of the model.

Applying the principles of the classical macroscopic continuum theory of crystalline solids with defects, we describe the inelastic behavior of the semi-crystalline polymer on the basis of the use of the defect density tensor. We introduce the term of defect density tensor in the effective medium of the pseudo-Euclidean four-dimensional space, as a generalization of defect definition in the three-dimensional continuum theory [35]. We define the tensor of defect density in the effective medium as a four-dimensional curl of the introduced four-dimensional distortion tensor, which is concerned with the potential and kinetic components of the mechanical energy of the material: $\alpha_{\nu \tau \gamma }=\left(\partial \beta_{\tau \gamma }/\partial x^{\nu }\right)-\left(\partial \beta_{\nu \gamma }/\partial x^{\tau }\right)$. The main difference between this tensor and the classical three-dimensional tensor (Equation (16)) takes into account the dynamic defects, including those caused by the non-laminar and non-stationary flow of amorphous (“soft”) phase. In this case, the definition and physical meaning of the spatial components α*_ijk_* completely correspond to the same in the classical three-dimensional continuum theory of defects in crystalline solids. Note that the 4D rotor (4-rotor) of the 4-tensor (Equation (19)) identically vanishes. Therefore, the defect density tensor is expressed in terms of the 4-tensor (Equation (20)), which is concerned with defects in crystalline and amorphous phases:

(21) $$\alpha_{\nu \tau \gamma }=\frac{\partial \beta_{\tau \gamma }^{\mathrm{int}}}{\partial x^{\nu }}-\frac{\partial \beta_{\tau \gamma }^{\mathrm{int}}}{\partial x^{\tau }}.$$

**Figure 1:**
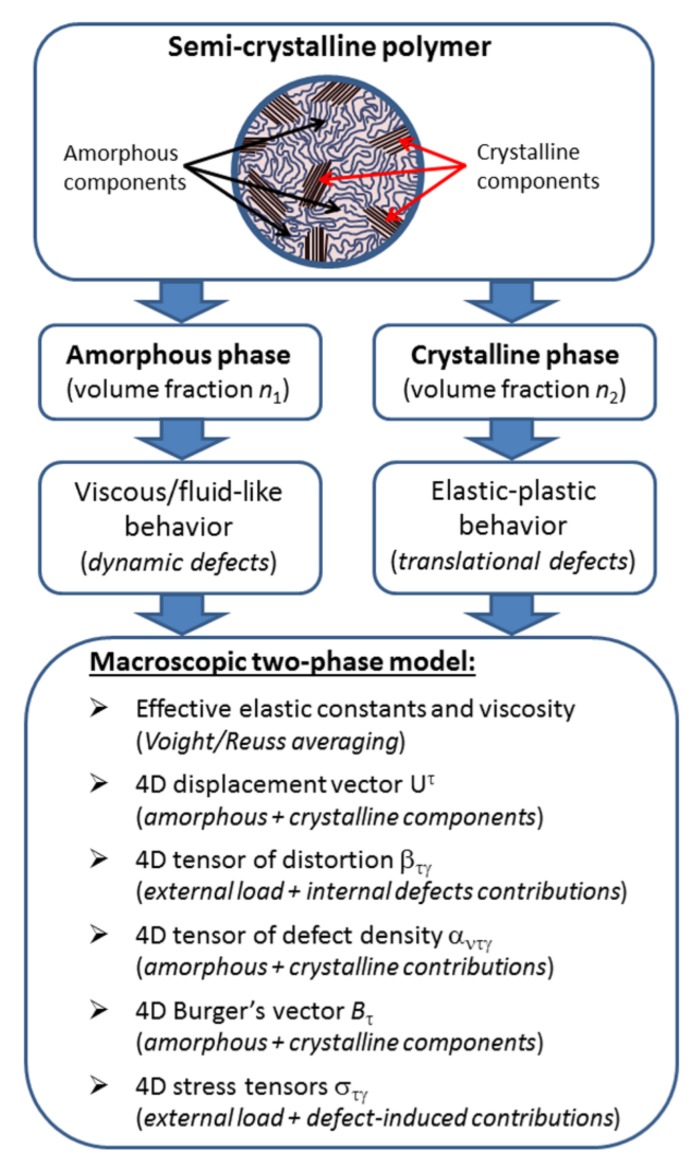
Schematic of the basic principles and main parameters of the developed two-phase model of semi-crystalline polymers.

The introduction of the concept of four-dimensional displacement vectors and distortion and defect density tensors leads to the necessity of building a generalized system of governing equations for semi-crystalline polymers. In comparison with the system of equations for crystalline solids, the new system should also include the equations of defect dynamics in the amorphous phase and the material relationships connecting these defects with the local stresses created by them. These relationships should take into account not only the interaction between defects in the amorphous phase, but also the interrelationship of translational defects in the crystalline phase and defects in the amorphous phase. We note that the tensor of local stress-caused defects and defect fluxes in the crystalline and amorphous components is also four-dimensional. Figure 1 schematically shows the basic principles and main parameters of the developed model.

## 3. Results and Discussion

In this section, we describe and discuss the main constituents of the developed model of the mechanical behavior of semi-crystalline polymers, which is based on the principles of the continuum theory of defects. Section 3.1 is devoted to the analysis and interpretation of the components of the 4-tensor of defect density. We show that the tensor of defect density can be represented as a superposition of two contributions: (1) dynamic defects in the amorphous phase and (2) translational defects and their fluxes in the crystalline phase. A four-dimensional (generalizing) formulation is proposed for Burger’s vector with a time component characterizing defects in the amorphous phase and spatial components characterizing defects in crystalline phase of the polymer. Section 3.2 presents the derivation of geometric and dynamic equations describing the behavior of defect fields in amorphous and crystalline components of the semi-crystalline polymer without taking into account the interaction between defects (in linear approximation). Section 3.3 describes the forces of interaction of defect fields: the self-action of defects, the interaction of defects of various types, and their interaction with external stress fields. Here, the energy and impulse characteristics of defect fields related with these interactions, as well as the interaction-induced internal stresses, are derived. Section 3.4 presents the full system of dynamic equations for defect fields in semi-crystalline polymers. This system takes into account the interaction between defects of the same type, as well as the interaction between defects of various types, and their interaction with the stress field caused by external loading. This is followed by a brief discussion of the advantages and limitations of the developed model (Section 3.5). Section 3.6 shows some simple examples of the application of the developed macroscopic continuum model for the analysis of natural vibrations of a defect field in the amorphous phase of a polymer, and general patterns of creep of polymers with a high degree of crystallinity under uniaxial loading.

### 3.1. Definition of Defects in a Semi-Crystalline Polymer as an Effective Medium

First of all, we have to define and classify defects in a semi-crystalline polymer. In the framework of the macroscopic continuum theory, defects are described integrally on the basis of the use of the 4-tensor of defect density α_ντγ_. Let us clarify the meaning of the components of this tensor. The components of the tensor α_ντγ_ with the zero value of the last index (γ = 0) characterize the defects in the amorphous phase (dynamic defects). The components of the tensor with spatial values of the last index (γ = 1, 2, 3) characterize the defects in the crystalline phase of the polymer.

Let us assume that γ = 0; then,

(22) $$\alpha_{\nu \tau 0}=\frac{\partial \beta_{\tau 0}^{\mathrm{int}}}{\partial x^{\nu }}-\frac{\partial \beta_{\nu 0}^{\mathrm{int}}}{\partial x^{\tau }}.$$

If ν = 0, then τ = *i* in Equation (21) can take only spatial indices since the defect density tensor is asymmetrical in the first two indices. In this case,

(23) $$\alpha_{0i0}=\frac{\partial \beta_{i0}^{\mathrm{int}}}{\partial x^{0}}-\frac{\partial \beta_{00}^{\mathrm{int}}}{\partial x^{i}}=\frac{1}{c^{2}}(\frac{\partial V_{i}}{\partial t}-\frac{\partial \Psi }{\partial x^{i}})\equiv \frac{1}{c^{2}}g_{i}=-\alpha_{i00}.$$

One can see that the introduced variable $\vec{g}$ has the physical meaning of acceleration. Therefore, the components α_0*i*0_ characterize the dynamic defects associated with the violation of the stationarity of the flow of the amorphous phase. Note that the parameter $\vec{g}$ is defined as a superposition of acceleration $\partial \vec{V}/\partial t$ and the gradient of potential Ψ.

Let us consider the case when ν and τ take spatial indices:

(24) $$\alpha_{ij0}=\frac{\partial \beta_{j0}^{\mathrm{int}}}{\partial x^{i}}-\frac{\partial \beta_{i0}^{\mathrm{int}}}{\partial x^{j}}=\frac{1}{c}\left(\frac{\partial V_{j}}{\partial x^{i}}-\frac{\partial V_{i}}{\partial x^{j}}\right).$$

We introduce the three-dimensional dual tensor,

(25) $${\tilde{\alpha }}_{k0}=\frac{1}{2}\Xi_{ijk}\alpha_{ij0}=\frac{1}{2}\Xi_{ijk}\frac{1}{c}\left(\frac{\partial V_{j}}{\partial x^{i}}-\frac{\partial V_{i}}{\partial x^{j}}\right)=\frac{1}{c}\omega_{k},$$

where ω*_k_* is the component of a vorticity vector $\vec{\omega }=\nabla \times \vec{V}$ (∇ is three-dimensional nabla operator), and $\Xi_{ijk}$ is Levi-Civita’s tensor. The feedback of tensors has the form

(26) $$\alpha_{ij0}=\Xi_{ijk}{\tilde{\alpha }}_{k0}=\frac{1}{c}\Xi_{ijk}\omega_{k}.$$

Thus, the components α*_ij_*_0_ are expressed through components of the pseudo-vector of vorticity, and characterize the dynamic defects associated with violation of laminar flow of the amorphous phase.

The index γ = *i* in Equation (21) takes spatial values; then,

(27) $$\alpha_{\nu \tau i}=\frac{\partial \beta_{\tau i}^{\mathrm{int}}}{\partial x^{\nu }}-\frac{\partial \beta_{\nu i}^{\mathrm{int}}}{\partial x^{\tau }}.$$

If the index ν = 0, then the index τ = *j* in Equation (4) can take only spatial values:

(28) $$\alpha_{0ji}=\frac{\partial \beta_{ji}^{\mathrm{int}}}{\partial x^{0}}-\frac{\partial \beta_{0i}^{\mathrm{int}}}{\partial x^{j}}=\frac{1}{c}\left(\frac{\partial \beta_{ji}^{\mathrm{int}}}{\partial t}-\frac{\partial v_{i}}{\partial x^{j}}\right)=\frac{1}{c}J_{ji}=-\alpha_{j0i}.$$

The expression in parentheses in Equation (28) determines the classical three-dimensional defects flux density tensor *J* (see Equation (17)). This tensor defines a three-dimensional (spatial) flux of translational defects in the crystalline component of the polymer. The dual tensor is related to the defect density tensor by the following relationship:

(29) $$\tilde{\alpha }=\nabla \times \beta^{el}=-\nabla \times \beta^{pl}.$$

Recall that β*^el^* and β*^pl^* are three-dimensional elastic and plastic distortions caused by defects in the crystalline component. Thus, the components α_0*ji*_ characterize the fluxes of translational defects in the crystalline phase.

Let us consider the components of the defect density tensor, whose indices are all spatial:

(30) $$\alpha_{kji}=\frac{\partial \beta_{ji}^{\mathrm{int}}}{\partial x^{k}}-\frac{\partial \beta_{ki}^{\mathrm{int}}}{\partial x^{j}}.$$

The dual tensor is written in the following way:

(31) $${\tilde{\alpha }}_{ni}=\frac{1}{2}\Xi_{kjn}\alpha_{kji}=\Xi_{nkj}\frac{\partial \beta_{ji}^{\mathrm{int}}}{\partial x^{k}}.$$

This corresponds to the classical definition of the defect density tensor in the three-dimensional continuum defects theory. The tensor α*_mpi_* is defined in terms of the dual tensor in the following way:

(32) $$\alpha_{mpi}=\Xi_{mpn}{\tilde{\alpha }}_{ni}.$$

Thus, the spatial components of the 4-tensor of defect density characterize the density of translational-type defects in the crystalline phase.

**Figure 2:**
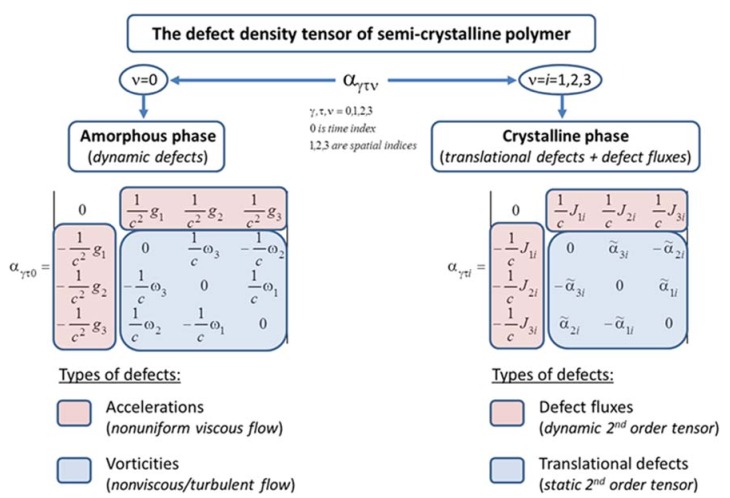
Contributions to the defect density tensor from defects in amorphous and crystalline components of a semi-crystalline polymer.

The results attained above can be presented in the form of two matrices (Figure 2). The first matrix is for the components of the density tensor of dynamic defects in the amorphous phase with the last time index. Taking into account Equations (23) and (26), it has the following form:

(33) $$\alpha_{\gamma \tau 0}=\left|\begin{array}{cccc} 0 & \frac{1}{c^{2}}g_{1} & \frac{1}{c^{2}}g_{2} & \frac{1}{c^{2}}g_{3}\\ -\frac{1}{c^{2}}g_{1} & 0 & \frac{1}{c}\omega_{3} & -\frac{1}{c}\omega_{2}\\ -\frac{1}{c^{2}}g_{2} & -\frac{1}{c}\omega_{3} & 0 & \frac{1}{c}\omega_{1}\\ -\frac{1}{c^{2}}g_{3} & \frac{1}{c}\omega_{2} & -\frac{1}{c}\omega_{1} & 0 \end{array}\right|.$$

The second matrix is for the components of the 4-tensor of the defect density in the solid (crystalline) phase with the last spatial index. Taking into account Equations (28) and (32), it is written in the following form:

(34) $$\alpha_{\gamma \tau i}=\left|\begin{array}{cccc} 0 & \frac{1}{c}J_{1i} & \frac{1}{c}J_{2i} & \frac{1}{c}J_{3i}\\ -\frac{1}{c}J_{1i} & 0 & {\tilde{\alpha }}_{3i} & -{\tilde{\alpha }}_{2i}\\ -\frac{1}{c}J_{2i} & -{\tilde{\alpha }}_{3i} & 0 & {\tilde{\alpha }}_{1i}\\ -\frac{1}{c}J_{3i} & {\tilde{\alpha }}_{2i} & -{\tilde{\alpha }}_{1i} & 0 \end{array}\right|.$$

We define Burger’s vector of a linear defect in a semi-crystalline polymer by integrating the distortion tensor over a closed spatial curve:

(35) $${\vec{B}}_{\tau }=\oint dx_{i}\cdot \beta_{i\tau }^{\mathrm{int}}.$$

The time component of Burger’s vector (τ = 0) is defined as follows:

(36) $${\vec{B}}_{0}=\frac{1}{c}\oint dx_{i}\cdot \beta_{i0}^{\mathrm{int}}=\frac{1}{c}\oint dx_{i}V_{i}=\frac{1}{c}\Gamma ,$$

where Γ is velocity circulation along a closed contour. One can see that the time (dynamic) component of Burger’s vector characterizes defects in the amorphous phase.

The spatial components of Burger’s vector are defined as

(37) $${\vec{B}}_{j}=\oint dx_{i}\cdot \beta_{ij}^{\mathrm{int}}=\oint dx_{i}\cdot \beta_{ij}^{el}=b_{j},$$

where *b_j_* are the spatial components of the discontinuity jump, which are characteristic of dislocation. Therefore, the spatial components characterize defects in the crystalline phase of polymer.

Thus, in the viscous (amorphous) phase of polymer, the “vorticity” $\vec{\omega }$ fields and “acceleration” $\vec{g}$ fields can be formed, which are dynamic defects by definition. In the solid (crystalline) phase of the polymer, translational defects fields are formed, which are defined in terms of the defect density tensor α and the defect flux density tensor *J*. A linear defect in the semi-crystalline polymer has a time (dynamic) component and three spatial components, $\vec{B}=\left(\Gamma /c,b_{i}\right)$.

### 3.2. Geometric and Dynamic Equations of the Defect Fields

The defect field stress tensor satisfies the following identical equation:

(38) $$\frac{\partial \alpha_{\gamma \tau \nu }}{\partial x^{\mu }}+\frac{\partial \alpha_{\tau \mu \nu }}{\partial x^{\gamma }}+\frac{\partial \alpha_{\mu \gamma \nu }}{\partial x^{\tau }}=0.$$

Note that intermediate algebraic passages of the derivation of this expression, as well as some subsequent relationships in the paper, are cumbersome; they are provided in Supplementary Materials.

Taking into account Matrices (33) and (34), this identity gives the equations below. We consider ν = 0. Taking into consideration Matrix (33), we obtain two equations:

(39) $$\nabla \times \vec{g}=\frac{\partial \vec{\omega }}{\partial t}\;\mathrm{and}\;\nabla \cdot \vec{\omega }=0.$$

Let ν = *i* take spatial indices; in this case, taking into consideration Matrix (34), we obtain the other two equations:

(40) $$\nabla \cdot \tilde{\alpha }=0\;\mathrm{and}\;\nabla \times J=\frac{\partial \tilde{\alpha }}{\partial t}.$$

Equation (40) are the basic geometric laws or field equations of the three-dimensional continuum defects theory. The obtained geometric or kinematic equations (Equations (39) and (40)) are suitable for any media within the framework of the proposed model, since they describe the motion of defects without taking into account the specific properties of the materials and the causes of defect motion.

To take into account the properties of materials and the causes of the defect motion, we proceed from the classical field theory [40]. In the classical field theory, the defect field stress tensor must satisfy the equation

(41) $$\frac{\partial \alpha_{\gamma \tau \nu }}{\partial x_{\gamma }}=\frac{1}{S}\sigma_{\tau \nu },$$

where the source of the defect field is on the right side, and *S* is the constant introduced to match the dimensions in the left and right sides of Equation (41). The four-dimensional stress tensor σ is considered as a source. The stresses σ_τν_ are caused by external loading. It follows from Equation (41) that the 4-stress tensor must satisfy the condition

(42) $$\frac{\partial \sigma_{\tau \nu }}{\partial x_{\tau }}=0.$$

The conditions of continuity and the motion equation for the material must follow from Equation (42).

At ν = 0, Equation (42) takes the form

(43) $$-\frac{\partial \sigma_{00}}{c\partial t}+\frac{\partial \sigma_{10}}{\partial x_{1}}+\frac{\partial \sigma_{20}}{\partial x_{2}}+\frac{\partial \sigma_{30}}{\partial x_{3}}=0.$$

In order for this expression to correspond to the continuity condition, it should be assumed that

(44) $$\sigma_{00}=-c^{2}\rho ,\;\sigma_{i0}=\sigma_{0i}=c\rho (V_{i}-\frac{\partial \Phi }{\partial x_{i}}+v_{i}+\frac{\partial u_{i}}{\partial t})\equiv c\rho {\vec{V}}^{*}.$$

Substituting these values in the expression above, we obtain the equation of continuity:

(45) $$\frac{\partial \rho }{\partial t}+\nabla \cdot \rho {\vec{V}}^{*}=0.$$

If ν takes spatial indices, Equation (42) is written as follows:

(46) $$-\frac{\partial \sigma_{0i}}{c\partial t}+\frac{\partial \sigma_{1i}}{\partial x_{1}}+\frac{\partial \sigma_{2i}}{\partial x_{2}}+\frac{\partial \sigma_{3i}}{\partial x_{3}}=0.$$

We define the component σ_0*i*_ in such a way that the resulting expression represents the motion equation for continuum. In this case, the spatial components of the 4-stress tensor should be considered as components of a three-dimensional stress tensor, the form of which are determined later. As a result, we obtain the motion equation

(47) $$\nabla \cdot \sigma =\frac{\partial }{\partial t}\rho {\vec{V}}^{*}.$$

Proceeding from the above, the components of the 4-stress tensor should be represented in the following way:

(48) $$\sigma_{\tau \nu }=\left|\begin{array}{cccc} -c^{2}\rho & c\rho V_{1}^{*} & c\rho V_{2}^{*} & c\rho V_{3}^{*}\\ c\rho V_{1}^{*} & \sigma_{11} & \sigma_{12} & \sigma_{13}\\ c\rho V_{2}^{*} & \sigma_{21} & \sigma_{22} & \sigma_{23}\\ c\rho V_{3}^{*} & \sigma_{31} & \sigma_{32} & \sigma_{33} \end{array}\right|.$$

We now turn to the construction of dynamic equations using Equation (18) and an expression for the 4-stress tensor (Equation (48)). We consider Equation (41) for ν = 0:

(49) $$-\frac{\partial \alpha_{0\tau 0}}{c\partial t}+\frac{\partial \alpha_{1\tau 0}}{\partial x_{1}}+\frac{\partial \alpha_{2\tau 0}}{\partial x_{2}}+\frac{\partial \alpha_{3\tau 0}}{\partial x_{3}}=\frac{1}{S}\sigma_{\tau 0}.$$

If τ = 0, then, from the last expression, we get

(50) $$\frac{\partial \alpha_{100}}{\partial x_{1}}+\frac{\partial \alpha_{200}}{\partial x_{2}}+\frac{\partial \alpha_{300}}{\partial x_{3}}=\frac{1}{S}\sigma_{00}.$$

Taking into account Matrices (33) and (48), from the latter expression, we obtain the equation for the field of “accelerations” in the amorphous phase:

(51) $$\nabla \cdot \vec{g}=\frac{c^{4}}{S}\rho .$$

Let τ = *i* take spatial indices (e.g., *i* = 1); then, we get

(52) $$-\frac{\partial \alpha_{010}}{c\partial t}+\frac{\partial \alpha_{210}}{\partial x_{2}}+\frac{\partial \alpha_{310}}{\partial x_{3}}=\frac{1}{S}\sigma_{10}.$$

Substituting the values from Matrix (33), we obtain

(53) $$-\frac{1}{c^{3}}\frac{\partial g_{1}}{\partial t}-\frac{1}{c}\frac{\partial \omega_{3}}{\partial x_{2}}+\frac{1}{c}\frac{\partial \omega_{2}}{\partial x_{3}}=\frac{1}{S}\sigma_{10}.$$

For other values *i* = 2, 3, we get similar expressions. Let us combine these three expressions:

(54) $$\nabla \times \vec{\omega }=-\frac{1}{c^{2}}\frac{\partial \vec{g}}{\partial t}-\frac{c^{2}}{S}\rho {\vec{V}}^{*}.$$

This is the equation for the field of “vorticities” in the amorphous phase.

Taking the divergence from Equation (54) and taking into account Equation (51), we obtain the continuity equation. If we assume that the index ν takes spatial values and τ = 0 in Equation (41), we obtain an equation for the tensor of translational defect flux density in the crystalline phase:

(55) $$\nabla \cdot J=-\frac{c^{2}}{S}\rho {\vec{V}}^{*}.$$

When the indices ν and τ in Equation (41) take only spatial values, the equation for the translational defect density tensor in the crystalline phase follows:

(56) $$\nabla \times \tilde{\alpha }=-\frac{1}{c^{2}}\frac{\partial J}{\partial t}-\frac{1}{S}\sigma .$$

We now combine geometric equations (Equations (39) and (40)) and dynamic equations (Equations (51), (54)–(56)) describing the behavior of defects in amorphous and crystalline components of the semi-crystalline polymer in the linear approximation:

(57a) $$\nabla \cdot \vec{\omega }=0,$$

(57b) $$\nabla \times \vec{g}=\frac{\partial \vec{\omega }}{\partial t},$$

(57c) $$\nabla \times \vec{\omega }=-\frac{1}{c^{2}}\frac{\partial \vec{g}}{\partial t}-\frac{1}{S}c^{2}\rho {\vec{V}}^{*},$$

(57d) $$\nabla \cdot \vec{g}=\frac{c^{4}}{S}\rho ,$$

(57e) $$\nabla \cdot \tilde{\alpha }=0,$$

(57f) $$\nabla \times J=\frac{\partial \tilde{\alpha }}{\partial t},$$

(57g) $$\nabla \cdot J=-\frac{c^{2}}{S}\rho {\vec{V}}^{*},$$

(57h) $$\nabla \times \tilde{\alpha }=-\frac{1}{c^{2}}\frac{\partial J}{\partial t}-\frac{1}{S}\sigma .$$

Equation (57) do not take into account the self-action of defects of the same type, as well as the interaction between defects of various types, and their interaction with stress field σ caused by external loading. In the next section, we present the relationships describing such interactions and obtain a system of coupled nonlinear equations describing the dynamics of defects in semi-crystalline polymers.

### 3.3. Interaction between the Defect Fields and the 4D Stress Tensor

We consider the interaction of defect fields with the 4D stress (4-stress) tensor σ separately for fields of “accelerations–vorticities” and fields of translational defects. Let us choose the expression for the interaction force of the defect fields with stresses in such a way that we can obtain physically meaningful results, which would partially coincide with the results of other authors:

(58) $$F^{\nu }=\sigma^{\tau \gamma }\alpha_{\tau \gamma }^{\nu }.$$

#### 3.3.1. Interaction Force between the Field of “Accelerations–Vorticities” and the 4-Stress Tensor

For the field of “accelerations–vorticities” in the amorphous component of the polymer (see Equation (33)), the equation for the force of interaction of fields is defined as follows:

(59) $$F^{\nu }=\sigma^{\tau 0}\alpha_{\tau 0}^{\nu }.$$

Taking into account Matrices (33) and (48), the time component of the force is defined as follows:

(60) $$F^{0}=\frac{\rho }{c}{\vec{V}}^{*}\cdot \vec{g}.$$

We substitute the value $\rho {\vec{V}}^{*}$ from Equation (57c) in this expression and add the zero term:

(61) $$\frac{S}{c^{3}}\left(\nabla \times \vec{g}-\frac{\partial \vec{\omega }}{\partial t}\right)\cdot \vec{\omega }=0.$$

The expression for the time component of the interaction force takes the form

(62) $$F^{0}=-\frac{S}{c^{3}}\nabla \cdot \left(\frac{S}{c^{2}}\vec{\omega }\times \vec{g}\right)-\frac{\partial }{c\partial t}\frac{S}{2}\left(\frac{1}{c^{4}}g^{2}+\frac{1}{c^{2}}\omega^{2}\right).$$

This expression implies the energy conservation law in a differential form:

(63) $$\frac{\partial }{\partial t}\frac{S}{2}\left(\frac{1}{c^{4}}g^{2}+\frac{1}{c^{2}}\omega^{2}\right)=-\nabla \cdot \left(\frac{S}{c^{2}}\vec{\omega }\times \vec{g}\right)-\rho {\vec{V}}^{*}\cdot \vec{g}.$$

On the left side of Equation (63), there is a change in the energy density of the dynamic defect field, and, on the right side, there is the divergence of the energy flux and a product of the material impulses and acceleration. Equation (63) shows that the energy of dynamic defect field in an elementary volume changes due to the flow of dynamic defects (and associated mechanical energy) across the volume boundary and the interaction of the field of accelerations with material impulses in this volume. The expression for the energy density of the acceleration and vorticity field is

(64) $$E^{\left(1\right)}=\frac{S}{2}\left(\frac{1}{c^{4}}g^{2}+\frac{1}{c^{2}}\omega^{2}\right),$$

and that for the energy flux through the unit surface is

(65) $${\vec{q}}^{\left(1\right)}=\frac{S}{c^{2}}\vec{\omega }\times \vec{g}.$$

Taking into account Matrix (33), the contribution of spatial components to the interaction force is defined as follows:

(66) $$\vec{F}=\rho \vec{g}+\vec{\omega }\times \rho {\vec{V}}^{*}.$$

In fact, this expression is a formulation of the classical Newton–Euler law for unit volume.

We express the density and impulse from the field (Equation (57c,d)) and substitute into the expression for the spatial force (Equation (66)):

(67) $$\vec{F}=\frac{S}{c^{4}}(\nabla \cdot \overset{\leftarrow }{g})\vec{g}-\vec{\omega }\times \left(\frac{S}{c^{2}}\nabla \times \vec{\omega }+\frac{S}{c^{4}}\frac{\partial \vec{g}}{\partial t}\right).$$

We take into account that $\vec{\omega }\times \nabla \times \vec{\omega }=\frac{1}{2}\nabla \omega^{2}-\vec{\omega }\cdot (\nabla \vec{\omega })$, in this case,

(68) $$\vec{F}=\frac{S}{c^{4}}(\nabla \cdot \overset{\leftarrow }{g})\vec{g}-\frac{S}{2c^{2}}\nabla \omega^{2}+\frac{S}{c^{2}}\vec{\omega }\cdot (\nabla \vec{\omega })-\frac{S}{c^{4}}\vec{\omega }\times \frac{\partial \vec{g}}{\partial t}.$$

We consider two expressions identically equal to zero:

(69) $$\frac{S}{c^{4}}\left(-\frac{\partial \vec{\omega }}{\partial t}+\nabla \times \vec{g}\right)\times \vec{g}=-\frac{S}{c^{4}}\frac{\partial \vec{\omega }}{\partial t}\times \vec{g}-\frac{S}{2c^{4}}\nabla g^{2}+\frac{S}{c^{4}}g\cdot (\nabla \vec{g})\equiv 0,\;\frac{S}{c^{2}}\vec{\omega }(\nabla \cdot \vec{\omega })\equiv 0.$$

We add these terms to the expression for the force, and taking into account the relationship $\nabla \cdot (\vec{g}\vec{g})=(\nabla \cdot \vec{g})\vec{g}+g\cdot (\nabla \vec{g})$, we find that the force acting per unit volume is determined as follows:

(70) $$\vec{F}=\nabla \cdot S\left(\frac{1}{c^{4}}\vec{g}\vec{g}+\frac{1}{c^{2}}\vec{\omega }\vec{\omega }-\frac{\delta }{2}(\frac{1}{c^{4}}g^{2}+\frac{1}{c^{2}}\omega^{2})\right)-\frac{\partial }{\partial t}(\frac{S}{c^{4}}\vec{\omega }\times \vec{g}),$$

where δ is a three-dimensional unit tensor.

If the spatial force is zero, we obtain the equation of motion for the free “acceleration–vorticity” fields (here, the term “free” implies the defect field that interacts neither with the sources of defects nor with defect fields of other types):

(71) $$\nabla \cdot S\left(\frac{1}{c^{4}}\vec{g}\vec{g}+\frac{1}{c^{2}}\vec{\omega }\vec{\omega }-\frac{\delta }{2}(\frac{1}{c^{4}}g^{2}+\frac{1}{c^{2}}\omega^{2})\right)=\frac{\partial }{\partial t}(\frac{S}{c^{4}}\vec{\omega }\times \vec{g}).$$

On the left side of Equation (71), under the divergence sign, there are stresses caused by the fields of accelerations and vorticities:

(72) $$\sigma^{\left(1\right)}=S\left(\frac{1}{c^{4}}\vec{g}\vec{g}+\frac{1}{c^{2}}\vec{\omega }\vec{\omega }-\frac{\delta }{2}(\frac{1}{c^{4}}g^{2}+\frac{1}{c^{2}}\omega^{2})\right).$$

On the right side of Equation (71), there is the field pulse under the time derivative:

(73) $${\vec{p}}^{\left(1\right)}=\frac{S}{c^{4}}\vec{\omega }\times \vec{g}.$$

Equations (65) and (73) show that the energy flux vector and impulse are related by

(74) $${\vec{q}}^{\left(1\right)}=c^{2}{\vec{p}}^{\left(1\right)}.$$

It is seen that the field of dynamic defects in the amorphous component of the semi-crystalline polymer has the basic physical characteristics of material objects, namely momentum and energy. Therefore, it is logical to introduce inertial characteristics of this field (mass). We can define the field mass, as the defect energy divided by velocity. In this case, the density of the field of “accelerations–vorticities” (in the amorphous component) is defined as

(75) $$\rho^{\left(1\right)}=\frac{S}{2c^{2}}\left(\frac{1}{c^{4}}g^{2}+\frac{1}{c^{2}}\omega^{2}\right).$$

Thus, the interaction of the defect field in the amorphous component of the polymer with the 4-stress tensor caused by external load leads to the appearance of internal stresses (field stresses) σ^(1)^. One can see that the field of defects in the amorphous phase of semi-crystalline polymers has its own mass, energy, impulse, and stresses. Consequently, this defect field is a self-dependent object (subsystem).

#### 3.3.2. Interaction Force between the Translational Defect Fields and the 4-Stress Tensor

For the defect fields of the translational type in the crystalline component of the polymer (see Equation (34)) the forces of interaction with the 4-stress tensor are determined as follows:

(76) $$F^{\gamma }=\sigma^{\tau i}\alpha_{\tau i}^{\gamma }.$$

Considering Matrix (34), the expression for the time component of the force is determined as follows:

(77) $$F^{0}=-\frac{1}{c}\sigma_{ki}J_{ki}.$$

Taking into account the definition of the defect flux density tensor (Equation (10)), the last expression can be rewritten as follows:

(78) $$F^{0}=\frac{1}{c}\sigma_{ki}\frac{\partial \beta_{ki}^{pl}}{\partial t}.$$

It is clear that the time component of the force has a meaning of the rate of dissipation of the energy of translational defects divided by velocity parameter *c*. We place σ into Equation (77) from the field Equation (57h) and, taking into account the equality $\left(\nabla \times J-\frac{\partial \tilde{\alpha }}{\partial t}\right)\cdot \cdot \tilde{\alpha }=0$, we get

(79) $$F^{0}=\frac{S}{2c}\frac{\partial }{\partial t}\left(\frac{1}{c^{2}}J^{2}+\alpha^{2}\right)+\frac{S}{c}\nabla \cdot (\alpha \times \cdot J).$$

Here, the mathematical symbol “··” denotes the double scalar convolution of tensors, and the symbol “×⋅” denotes the vector product of tensors with respect to the first indices, and the scalar product with respect to the second indices.

Taking into account the definition of the defect flux density tensor (Equation (17)), the last expression can be rewritten as follows:

(80) $$\frac{\partial }{\partial t}\frac{S}{2}\left(\frac{1}{c^{2}}J^{2}+\alpha^{2}\right)=\sigma \cdot \cdot \frac{\partial \beta^{pl}}{\partial t}-\nabla \cdot S(\alpha \times \cdot J).$$

We integrate the obtained expression over a certain volume *W*,

(81) $$\frac{\partial }{\partial t}\int \frac{S}{2}\left(\frac{1}{c^{2}}J^{2}+{\tilde{\alpha }}^{2}\right)dW=\int \sigma \cdot \cdot \frac{\partial \beta^{pl}}{\partial t}dW-\oint d\vec{s}\cdot S(\tilde{\alpha }\times \cdot J),$$

and we find that time variation in the energy of the field of translational defects in the volume W is due to the scattered work of stresses on the translational defect fluxes and the energy flow through the closed surface *s*, which bounds the volume. The expression for the translational defect energy density has the following form:

(82) $$E^{\left(2\right)}=\frac{S}{2}\left(\frac{1}{c^{2}}J^{2}+{\tilde{\alpha }}^{2}\right).$$

The expression for energy flow is as follows:

(83) $${\vec{q}}^{\left(2\right)}=S(\tilde{\alpha }\times \cdot J).$$

Spatial force is defined as

(84) $$\vec{F}=(\sigma \times \cdot \tilde{\alpha })+\rho J\cdot {\vec{V}}^{*}.$$

The spatial contribution to the force (3D vector) includes two components: static (interaction of the field of translational defects with external stresses) and dynamic (interaction of the defect flow and the flow of the material).

We place σ and $\rho {\vec{V}}^{*}$ in Equation (84) from Equation (57g,h):

(85) $$\vec{F}=-S\left(\nabla \times \alpha +\frac{1}{c^{2}}\frac{\partial J}{\partial t}\right)\times \cdot \alpha -\frac{S}{c^{2}}J\cdot (\nabla \cdot J).$$

Let us add two zero terms to the right side of the expression for force,

(86) $$\left(\nabla \times J-\frac{\partial \alpha }{\partial t}\right)\times \cdot J=0,\;\tilde{\alpha }\cdot (\nabla \cdot \tilde{\alpha })=0,$$

and take into account the following identities written in the coordinate representation:

(87) $${\left((\nabla \times J)\times \cdot J\right)}_{i}=\frac{\partial J_{ij}}{\partial x_{k}}J_{kj}-\frac{\partial }{\partial x_{i}}J_{jk}J_{jk},\;{\left(\nabla \cdot (J\cdot J)\right)}_{i}=\frac{\partial J_{jk}}{\partial x_{j}}J_{ik}+J_{jk}\frac{\partial J_{ik}}{\partial x_{j}}.$$

In this case, the expression for the spatial force is finally written as follows:

(88) $$\vec{F}=-S\nabla \cdot \left(\frac{1}{c^{2}}J\cdot J+\tilde{\alpha }\cdot \tilde{\alpha }\right)+\frac{S}{2}\left(\frac{1}{c^{2}}\nabla J^{2}+\nabla {\tilde{\alpha }}^{2}\right)+\frac{S}{c^{2}}\frac{\partial }{\partial t}\tilde{\alpha }\times \cdot J.$$

If the spatial components of the interaction force are zero, then Equation (88) is the motion equation of free fields of the translational defects:

(89) $$\nabla \cdot S\left\{\frac{1}{c^{2}}J\cdot J+\tilde{\alpha }\cdot \tilde{\alpha }-\frac{1}{2}\delta (\frac{1}{c^{2}}J^{2}+{\tilde{\alpha }}^{2})\right\}=\frac{S}{c^{2}}\frac{\partial }{\partial t}\tilde{\alpha }\times \cdot J.$$

On the left side, there is a three-dimensional field stress tensor under the divergence sign,

(90) $$\sigma^{\left(2\right)}=S\left\{\frac{1}{c^{2}}J\cdot J+\tilde{\alpha }\cdot \tilde{\alpha }-\frac{1}{2}\delta (\frac{1}{c^{2}}J^{2}+{\tilde{\alpha }}^{2})\right\},$$

and, on the right side, there is the field impulse of translational defects,

(91) $${\vec{p}}^{\left(2\right)}=\frac{S}{c^{2}}\tilde{\alpha }\times \cdot J.$$

As in the case of defects in the amorphous component, the field of defects in the crystalline component of the semi-crystalline polymer has momentum and energy. Through analogy with Equation (75), we can define the density of the field of translational defects and defect fluxes in a crystalline component as

(92) $$\rho^{\left(2\right)}=\frac{S}{2c^{2}}\left(\frac{1}{c^{2}}J^{2}+{\tilde{\alpha }}^{2}\right).$$

Thus, the interaction of the field of defects in the crystalline component with the 4-stress tensor caused by the external load leads to the appearance of internal stresses (field stresses) σ^(2)^. The field translational defects and defect flows also have their own energy, momentum, and mass, that is, they can be considered as an independent subsystem.

**Figure 3:**
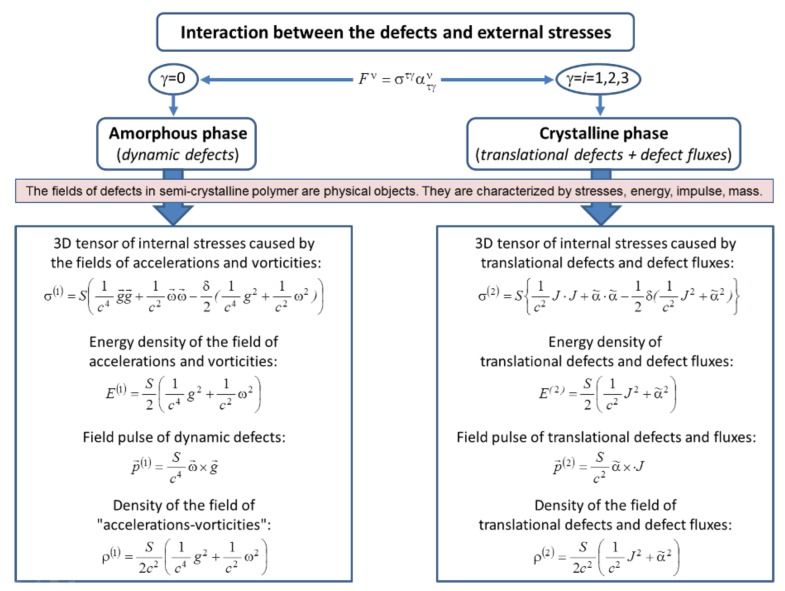
The expressions for the main characteristics of the defects as physical objects, which form the separate subsystem of a semi-crystalline polymer.

Figure 3 summarizes the main implications of the interaction between defects in the components of a semi-crystalline polymer and the stress field caused by the external load.

### 3.4. The System of Dynamic Equations for Defect Fields in Semi-Crystalline Polymers

When revising the system of Equation (57) describing the behavior of defects in amorphous and crystalline components of the semi-crystalline polymer, the above-discussed field characteristics (mass, impulse, and stresses) must be taken into account. In this case, the geometric Equation (57a,b,e,f) do not change. When rewriting dynamic equations, we should take into account that the fields of “accelerations–vorticities” have mass (Equation (75)), and the fields of translational defects have mass (Equation (92)). These masses enter into Equation (57d) with the volume fractions of solid (crystalline) and viscous (amorphous) components. Also, it is necessary to add the field impulses (Equation (73)) for the “accelerations–vorticity” field, and field impulses (Equation (91)) for the field of translational defects to Equation (57c,g) with the corresponding volume fractions of two phases. As a result, Equation (57a–h) take the following forms:

(93a) $$\nabla \cdot \vec{\omega }=0,$$

(93b) $$\nabla \times \vec{g}=\frac{\partial \vec{\omega }}{\partial t},$$

(93c) $$\nabla \times \vec{\omega }=-\frac{1}{c^{2}}\frac{\partial \vec{g}}{\partial t}-\frac{c^{2}}{S}\rho {\vec{V}}^{*}-n_{2}\frac{c^{2}}{S}{\vec{p}}^{\left(2\right)}-n_{1}\frac{c^{2}}{S}{\vec{p}}^{\left(1\right)},$$

(93d) $$\nabla \cdot \vec{g}=\frac{c^{4}}{S}\rho +n_{1}\frac{c^{4}}{S}\rho_{1}+n_{2}\frac{c^{4}}{S}\rho_{2},$$

(93e) $$\nabla \cdot \tilde{\alpha }=0,$$

(93f) $$\nabla \times J=\frac{\partial \tilde{\alpha }}{\partial t},$$

(93g) $$\nabla \cdot J=-\frac{c^{2}}{S}\rho {\vec{V}}^{*}-n_{2}\frac{c^{2}}{S}{\vec{p}}^{\left(2\right)}-n_{1}\frac{c^{2}}{S}{\vec{p}}^{\left(1\right)},$$

(93h) $$\nabla \times \tilde{\alpha }=-\frac{1}{c^{2}}\frac{\partial J}{\partial t}-\frac{1}{S}\sigma -\frac{1}{S}\sigma^{\left(1\right)}-\frac{1}{S}\sigma^{\left(2\right)}.$$

Here, the first four equations correspond to dynamic defects (accelerations and vorticities) in the amorphous phase, while the next four equations describe translational defects and defect fluxes in the crystalline phase. One can see from these equations that defects of various types do not behave independently and largely interdetermine their evolutions. Let us briefly describe the physical meaning of each of these equations.

- Equation (93a) implies that there are no sources and sinks of vorticity in the material (the vortex lines are closed or come to the surface);
- Equation (93b) defines the relationship between accelerations and vorticities in the amorphous phase of the polymer;
- Equation (93c) shows that vorticity is generated by a change in the acceleration of the amorphous phase, a material impulse, and field impulses of defects in amorphous and crystalline phases. This relationship describes the interaction between defects of various types, in particular, it shows that the fluxes of translational defects in the crystalline phase of the polymer can “generate” dynamic defects in the amorphous phase;
- Equation (93d) shows that the sources of accelerations in the amorphous phase are the material density and the densities of the defect fields in both phases. This equation also demonstrates the effect of the fields of translational defects and their fluxes in the crystalline phase on the nucleation of dynamic defects in the amorphous phase;
- Equation (93e) implies that translational defects in the crystalline phase are closed or exit to the surface;
- Equation (93f) defines the relationship between the translational defect flux and the density of translational defects;
- Equation (93g) shows that material impulses and field impulses of defects in the amorphous and crystalline phases cause the fluxes of translational defects. The relationship between defect fluxes in the crystalline and amorphous phases can be illustrated by combining the Equation (93c,g): $\nabla \cdot J=\nabla \times \vec{\omega }+\frac{1}{c^{2}}\frac{\partial \vec{g}}{\partial t}$. The meaning of the relationship is that dynamic defects in the amorphous phase (change in acceleration and/or curl of vorticity) can “generate” the motion (flux) of translational defects in the crystalline phase;
- Equation (93h) shows that defects of the translational type appear in (or disappear from) the crystalline component of some volume of the semi-crystalline polymer as a result of external stresses, and field stresses caused by defects in the amorphous and crystalline phases, as well as changes in the rate of flow of translational defects through the surface of this volume.

The desired variables of the system of Equation (93) are the densities of the four types of considered defect fields (accelerations $\vec{g}$ and vorticities $\vec{\omega }$ in the amorphous phase, tensor of the density of translational defects $\tilde{\alpha }$, and the tensor of the density of translational defects flux *J* in the crystalline phase—24 unknown parameters altogether). In the system of Equation (93), the basic (independent) equations are Equation (93b,c,f,h)—23 equations altogether.

Equation (93) must be supplemented by the material relationships that connect the following:

- Stresses σ and elastic displacements ${\vec{u}}^{el}$ caused by external loading (Hooke’s law);
- Elastic displacements ${\vec{u}}^{el}$ (or displacement rates) with the characteristics of defect fields in the crystalline phase.

Thus, the number of basic equations is equal to the number of unknown variables. In this case, the system is closed (complete) and can be solved numerically, when specifying the boundary and initial conditions for the fields of dynamic and translational defects. Together with boundary and initial conditions, the resulting equation system allows describing the deformation of a two-phase polymer material when one component is in the solid (crystalline) state, and the second one is in the viscous (amorphous) state.

Note that the system of Equation (93) can be written in terms of potentials. Indeed, recall that

(94) $$\vec{\omega }=\nabla \times \vec{V},\;\vec{g}=\frac{\partial \vec{V}}{\partial t}-\nabla \Psi ,\;\tilde{\alpha }=\nabla \times \beta^{el}=-\nabla \times \beta^{pl},\;J_{ji}=\frac{\partial \beta_{ji}^{\mathrm{int},el}}{\partial t}-\frac{\partial \nu_{i}}{\partial x^{j}}=-\frac{\partial \beta_{ji}^{\mathrm{int},pl}}{\partial t}.$$

Here, the potentials are the rate of viscous flow of the amorphous phase and the potential Ψ, as well as translational defect-related elastic or plastic distortions in the crystalline phase. These potentials can be used as the desired variables. Definition of the initial and boundary conditions for these parameters is much simpler and clearer than for defect densities. This makes the formulation of Equation (93) in terms of potentials more convenient for numerical implementation.

We also note that the solutions of the system of Equation (93) must satisfy the following conditions:

(1) The generalized continuity equation:

(95) $$\frac{\partial }{\partial t}(\rho +n_{1}\rho_{1}+n_{2}\rho_{2})+\nabla \cdot (\rho {\vec{V}}^{*}+n_{2}{\vec{p}}^{\left(2\right)}+n_{1}{\vec{p}}^{\left(1\right)})=0.$$

To obtain Equation (95), we take the divergence operation from Equation (93c) and take into account Equation (93d).

(2) The generalized motion equation for a semi-crystalline polymer material with defects:

(96) $$\nabla \cdot \left[\sigma +\sigma^{\left(1\right)}+\sigma^{\left(2\right)}\right]=\frac{\partial }{\partial t}\left[\rho {\vec{V}}^{*}+n_{2}{\vec{p}}^{\left(2\right)}+n_{1}{\vec{p}}^{\left(1\right)}\right].$$

To obtain Equation (96), we take the divergence from Equation (93h) and take into account Equation (93g).

### 3.5. Discussion

Within the framework of the developed continuum mechanical model, the semi-crystalline polymer is considered as a two-phase (two-component) elastic–viscous–plastic material. The first (amorphous) phase is treated as a viscous fluid. The second (crystalline) phase is regarded as an elastic–plastic crystalline material that contains translational (dislocation-type) defects. The elastic response of the polymer is described on the basis of the approach of an effective isotropic medium using effective (averaged) elastic constants and averaged elastic displacements/strains. The inelastic response of the polymer is described as a superposition of inelastic responses from the crystalline and amorphous phases. The carriers of inelastic deformation in both components are defects. Due to the difference in the rheological properties of the components, the defects in different components have different origins. In particular, the defects in the amorphous phase are local accelerations and vorticity, which characterize a violation of the stationarity of the viscous flow and the appearance of turbulence, respectively. Both types of defects are dynamic, and their contribution to the inelastic behavior of the material increases upon increasing the rate of external loading. The defects in the crystalline component of the semi-crystalline polymer are translational defects. These defects create internal static stress fields and distortions. Translational defects are able to move under the influence of both the external loading and internal stresses caused by defects. During the motion of translational defects, the fields of defect-induced elastic and plastic distortions change. Therefore, translational defect fluxes are considered as a separate (dynamic) type of defects in the crystalline phase. The contribution of these defects, as well as that of dynamic defects in the amorphous phase, to the behavior of the material increases with an increase in the external loading rate. Note that, since all types of defects listed above create internal stress fields, the evolution of defects (in particular, the change in the flux of translational defects in the crystalline component or the magnitude of vorticity or acceleration in the amorphous component) is also determined by the interaction of defects with internal stress fields. This causes the interdependence of the inelastic behavior of the amorphous and crystalline phases, thereby determining the strong nonlinearity of the response of a semi-crystalline polymer as a two-phase material.

Within the framework of the developed model, the defect fields in the crystalline and amorphous components are considered as physical objects and possess, in addition to energy, their own impulse and mass. The account of the energy and inertial characteristics of defects makes it possible to apply the model to study the dependence of the mechanical response of a semi-crystalline polymer on the magnitude of the applied load and on the loading rate.

The developed continuum model is macroscopic. Here, the term “macroscopic” implies that a mathematically infinitesimal volume of the medium corresponds to a physically small but finite volume that contains a sufficiently large number of defects for their integral/averaged description. Therefore, the description of defect ensembles in the crystalline and amorphous components is integral and is realized by means of a four-dimensional defect density tensor, which combines both dynamic and static defect-related components. We note that, unlike the microscopic models of individual defects in a crystal, the macroscopic model does not take into account singularities in the stress/strain fields from individual translational defects. The macroscopic model considers the averaged fields of distortions and stresses caused by the ensemble of defects. Averaging smoothens these fields, and they do not contain singularities (this is one of the results of the condition of continuity of the medium).

The formalism of the model implies that after removal of external loads (and completion of relaxation processes in the material), dynamic defects and associated stresses disappear; however, translational defects in the crystalline component and associated static internal stress fields do not disappear. These internal stresses are residual stresses. The specific form of the spatial distribution of the components of the residual stress tensor in the modeled polymer is determined from the loading history and is generally complex and inhomogeneous. Therefore, the inelastic behavior of the sample with residual stresses under repeated loading is, in general, anisotropic, despite the approximation of the isotropy of the macroscopic elastic characteristics of the polymer. The degree of anisotropy of inelastic behavior is determined by the features of the distribution and the magnitude of the residual stresses (i.e., the history of preloading), as well as the orientation of the applied repeated load. We note here that the inherent anisotropy of inelastic behavior of the polymer due to the static stress field from translational defects distinguishes the developed model from widely used classic models of plastic flow of solids (including polymers), where defect-caused internal stresses are not taken into account.

At the end of the discussion, it is necessary to mention the main limitations of the developed model. The first limitation is related to the description of the amorphous phase as a viscous-flowing matter. This approximation assumes the presence of only dynamic defects (accelerations and vorticity) in the amorphous phase and does not take into account the possibility of the existence of static defects creating static internal stress fields. Such static defects in the amorphous phase can be stable if the amorphous phase is in the glassy state. The second limitation of the model is due to the fact that the mechanical behavior of a semi-crystalline polymer is described on the basis of the approximation of a series connection of elastic and viscous elements describing the response of the crystalline and amorphous components, respectively (the Maxwell mechanical model). However, in general, the rheological properties of polymers (and the corresponding rheological model) can be more complex. As a third limitation, it is necessary to mention the use of the approximation of an effective isotropic medium. It should be noted that the potential of the mathematical basis of the model makes it possible to expand the field of its application to polymers with an anisotropic internal structure and anisotropic elastic behavior; however, this leads to a significant complication of the system of Equation (93).

### 3.6. Some Analytical Solutions for Amorphous and Crystalline Polymer Phases

In general, the use of a system of nonlinear interrelated Equation (93) to study various aspects of the mechanical behavior of semi-crystalline polymers can only be carried out on the basis of numerical (computer) modeling. The building of a numerical model based on the developed macroscopic continuum model is a separate task that goes beyond the scope of this article. Nevertheless, in some particular cases, the system of linear Equation (57), in which the relationship between the crystalline and amorphous phases is weak and manifests itself only through material density *ρ* and momentum $\rho {\vec{V}}^{*}$, can be solved analytically. As a rule, it is possible to obtain analytical solutions of particular problems only for individual components of the polymer. The present study is limited by the analysis of two particular problems, which allow analytical solutions.

#### 3.6.1. Analysis of the Natural Vibrations of the Defect Field in the Amorphous Phase of the Polymer

The semi-crystalline polymer is a two-phase material with different spatial regions occupied by a crystalline or amorphous phase. In the general case, the amorphous phase contains dynamic defects, which are described by the proposed macroscopic continuum model in terms of the fields of accelerations and vorticities. We showed above that these fields are physical objects characterized by their own energy, momentum, and density. Therefore, the fields of defects, like other mechanical objects, are also characterized by their natural (free) oscillations. It is clear that the modes of these oscillations depend, among other things, on the size of the spatial regions in the polymer occupied by the amorphous phase. In this section, we analyze the natural oscillations of the vorticity field in the amorphous phase, which occupies some bounded region of linear dimension *L* in the volume of the polymer sample. We recall that the natural oscillations are considered in the absence of external actions.

Within the approximation of a weak coupling between the fields of defects in the amorphous and crystalline phases (linear approximation, Equation (57)), the system of equations for the fields of defects in the amorphous phase takes the following form:

(97a) $$\nabla \cdot \vec{\omega }=0,$$

(97b) $$\nabla \times \vec{g}=\frac{\partial \vec{\omega }}{\partial t},$$

(97c) $$\nabla \times \vec{\omega }=-\frac{1}{c^{2}}\frac{\partial \vec{g}}{\partial t}-\frac{c^{2}\rho }{S}(\vec{V}-\nabla \Phi ),$$

(97d) $$\nabla \cdot \vec{g}=\frac{c^{4}}{S}\rho .$$

Using the example of the vorticity field, we show that the field of defects in a bounded volume of the amorphous polymer phase is characterized by a discrete spectrum of energy and vibration modes.

Upon applying the rotor operation to Equation (97c), we get

(98) $$\nabla (\nabla \cdot \vec{\omega })-\Delta \vec{\omega }=-\frac{1}{c^{2}}\frac{\partial }{\partial t}(\nabla \times \vec{g})-\frac{c^{2}\rho }{S}(\nabla \times \vec{V}),$$

where Δ is a Laplace operator.

Taking into account Equation (97a,b), and the definition of the vector of vorticity, we obtain from Equation (98) the linear Klein–Gordon equation for the field of vorticities:

(99) $$\Delta \vec{\omega }-\frac{1}{c^{2}}\frac{\partial^{2}\vec{\omega }}{\partial t^{2}}-\frac{c^{2}\rho }{S}\vec{\omega }=0.$$

One can see that this equation splits into three equations for the three components (ω*_x_*, ω*_y_*, and ω*_z_*).

For simplicity, we consider the solution of this equation for the component ω*_x_*. We assume that the linear size of the region occupied by the amorphous phase is equal to *L* along the *X*-axis of the coordinate system. Then, Equation (99) for the component ω*_x_* takes the following form:

(100) $$\frac{\partial^{2}\omega }{\partial x^{2}}=\frac{1}{c^{2}}\frac{\partial^{2}\omega }{\partial t^{2}}+m_{0}^{2}\omega .$$

Hereafter, a variable ω implies the component ω*_x_*, and we take into account its dependence only on the coordinate *x*. We also introduce the parameter *m*_0_ ($m_{0}^{2}=c^{2}\rho /S$), which has the dimension of the inverse square of length.

Note that Equation (100) can be obtained from the Lagrangian for the vorticity field,

(101) $$\text{E}=\frac{1}{2}{\left(\frac{\partial \omega }{\partial x}\right)}^{2}-\frac{1}{2}{\left(\frac{1}{c}\frac{\partial \omega }{\partial t}\right)}^{2}-\frac{m_{0}^{2}}{2}{\left(\omega \right)}^{2},$$

using the Lagrange–Euler equations,

(102) $$\frac{\partial Ε}{\partial \omega }-\frac{\partial }{\partial x}\frac{\partial Ε}{\partial \frac{\partial \omega }{\partial x}}-\frac{\partial }{\partial t}\frac{\partial Ε}{\partial \frac{\partial \omega }{\partial t}}=0.$$

The Hamiltonian or the energy of the vorticity field is defined as

(103) $$H=\frac{1}{2}{\left(\frac{\partial \omega }{\partial x}\right)}^{2}+\frac{1}{2}{\left(\frac{1}{c}\frac{\partial \omega }{\partial t}\right)}^{2}+\frac{m_{0}^{2}}{2}{\left(\omega \right)}^{2}.$$

Let us impose the condition of periodicity on the function ω in the direction of the *X*-axis: ω(x,t)=ω(x+L,t). In this case, the solution is sought as an expansion of the solution ω(*x*,*t*) of Equation (100) in a system of functions having a period and satisfying the orthonormal condition. We choose a system of orthonormal functions [41] such as

(104) $$f(x)=\sqrt{\frac{2}{L}}\cos\frac{2\pi nx}{L}.$$

Here, *n* is any integer of positive or negative numbers, including zero. In this case, the solution of Equation (100) is written as an expansion in the system of orthonormal functions (104):

(105) $$\omega (x,t)=\sum_{n}A_{n}(t)\cos\frac{2\pi nx}{L},$$

where $A_{n}(t)$ is a real amplitude. Substituting this solution into Equation (100), then multiplying by a fixed function $\sqrt{\frac{2}{l}}\cos\frac{2\pi px}{l}$ and integrating over *dx* from 0 to *L*, and taking into account the orthonormality condition,

(106) $$\frac{2}{L}\int_{0}^{L}\cos\frac{2\pi px}{L}\cos\frac{2\pi nx}{L}dx=\begin{array}{c} 0,\;p\neq n\\ 1,p=n \end{array},$$

we obtain an ordinary differential equation for amplitudes:

(107) $$\frac{d^{2}A_{p}(t)}{dt^{2}}+c^{2}K_{n}{}^{2}A_{p}(t)=0.$$

Equation (107) is an oscillator equation that oscillates with a frequency

(108) $$\nu_{n}=cK_{n}=c\sqrt{{\left(\frac{2\pi n}{L}\right)}^{2}+m_{0}^{2}},\;n=\;0,\;1,\;2,\;3\ldots$$

Having performed similar operations with Hamiltonian (103), we obtain its expression through the amplitudes

(109) $$H=\sum_{n}\left[{\left(\frac{dA_{n}(t)}{dt}\right)}^{2}+c^{2}K^{2}{\left(A_{n}(t)\right)}^{2}\right].$$

Hamiltonian (109) is the sum of Hamiltonians, of which each is the energy of a separate oscillator.

Thus, the field of vorticities in a bounded volume of the amorphous phase is characterized by the spectrum of natural frequencies, and this spectrum is discrete. Each mode of the natural oscillations of the vorticity field along a certain direction *X* can be associated with a linear oscillator. The component of the wave vector *K_n_* along this direction takes discrete values (Equation (108)). The energy of the vorticity field along the *X*-axis is the sum of the energies of oscillators, whereby each of them oscillates with a frequency ν*_n_* taking discrete values. It is important to note that the values of these frequencies depend, in addition to the physical parameters of the amorphous phase, on the linear size *L* of the region occupied by the amorphous phase along the *X*-axis. In this case, the oscillation energy of the vorticity field also takes discrete values. A similar study can be carried out for other components of the vorticity vector (ω*_y_*, ω*_z_*) provided that the amorphous phase is limited along the corresponding *Y*- and *Z*-axes. The expressions obtained in this way are similar to Equations (107)–(109).

The results of the analysis show that the natural oscillations of the vorticity field in the amorphous phase, which occupies a certain bounded region in the polymer, have a discrete characteristic. Each oscillation mode can be mapped to linear oscillators whose frequencies take discrete values. The energy of the vorticity field, in this case, is defined as the sum of the energies of individual oscillators.

A similar analysis can be easily carried out for the acceleration fields in the internal regions of the polymer occupied by the amorphous phase, as well as for the defect fields in the internal regions of the polymer occupied by the crystalline phase. The corresponding frequency and vibration energy spectra are discrete.

The result obtained is important for understanding the behavior of semi-crystalline polymers under conditions of dynamic and, in particular, high-frequency loading. If there is a predominant size of the polymer regions occupied by the amorphous phase, then, at external loading with a frequency coinciding with one of the lowest natural frequencies of the vorticity field, the amplitude and vibrational energy of oscillation of these dynamic defects can increase many times due to the resonance effect. Moreover, due to the interaction of defects of various types (see the system of Equation (93)), such oscillations inevitably excite oscillations of other components of the defective subsystem of the polymer (acceleration fields in the amorphous phase, and translational defects and their fluxes in the crystalline phase). The energies of these oscillations are determined by the energy of the oscillation of the vorticity field, and also by the volume fraction of the amorphous phase. A similar result is achieved if the frequency of external load corresponds to one of the natural frequencies of defects of another type. Thus, the resonant excitation of oscillations of one type of defect in the semi-crystalline polymer is able to cause excitation of oscillations of the entire defect system and can qualitatively change the characteristic of the inelastic behavior of the material as a whole. A detailed study of the excitation of oscillations of the entire defect system of polymers upon the resonant excitation of natural oscillations of defects of any type is the subject of a separate paper.

#### 3.6.2. Analysis of General Patterns of Creep of Polymers with a High Degree of Crystallinity under Uniaxial Loading

As a simple example showing the varied ability of the developed model, we considered the creep phenomenon for the particular case of polymers with a high degree of crystallinity. The influence of the amorphous component on the behavior of the material can be neglected for this kind of polymer (*n*_1_ = 0).

In this case, the system of Equation (93e–h), describing the crystalline polymer in the absence of the amorphous phase, is written in the following form:

(110a) $$\nabla \cdot \tilde{\alpha }=0,$$

(110b) $$\nabla \times J=\frac{\partial \tilde{\alpha }}{\partial t},$$

(110c) $$\nabla \cdot J=-\frac{c^{2}}{S}\rho (\vec{v}+\frac{\partial \vec{u}}{\partial t})-\frac{c^{2}}{S}{\vec{p}}^{\left(2\right)},$$

(110d) $$\nabla \times \tilde{\alpha }=-\frac{1}{c^{2}}\frac{\partial J}{\partial t}-\frac{1}{S}\sigma -\frac{1}{S}\sigma^{\left(2\right)}.$$

We consider the uniaxial loading along the *x*-axis, when only α*_xx_* and *J_xx_* components are non-zero. These components depend on the *x*-coordinate only. This simple case allows the derivation of analytical solutions.

In this case, the defect density tensor vanishes by virtue of its definition, and only Equation (110d) remains from the system of Equation (110a–d). This equation takes the form

(111) $$\frac{1}{c^{2}}\frac{\partial J_{xx}}{\partial t}+\frac{1}{S}\sigma_{xx}+\frac{1}{2c^{2}}J_{xx}^{2}=0.$$

In this equation, the defect flux density tensor has only one non-zero component: $J_{xx}=-\partial \beta_{xx}^{pl}/\partial t=-\partial \varepsilon /\partial t=-\dot{\varepsilon }$, where ε is an axial plastic strain. We suppose that the stresses σ are caused by an external load. Such stresses include stresses from mechanical σ*^ext^* and thermal $\sigma^{\left(T\right)}=K\theta (T-T_{0})\delta$ loads, where *K* and θ are the bulk modulus and the coefficient of thermal expansion, respectively, and *T* is the absolute temperature. We also take into account the stresses due to the viscous motion of translational defects $\sigma^{\left(J\right)}=\eta J=-\eta \dot{\varepsilon }$, where η is the dynamic viscosity coefficient. In the considered problem, the σ stress tensor has one non-zero component σ*_xx_* (it reduces to a scalar). Furthermore, we omitted the subscript “*xx*” for simplicity. In this case, stresses σ in Equation (110d) are written as

(112) $$\sigma =\sigma^{ext}+\sigma^{\left(T\right)}+\sigma^{\left(J\right)}=\sigma^{ext}+K\theta (T-T_{0})+\eta J,$$

and Equation (111) takes the form

(113) $$\frac{1}{c^{2}}\ddot{\varepsilon }-\frac{1}{S}\sigma^{ext}-\frac{1}{S}K\theta (T-T_{0})+\frac{\eta }{S}\dot{\varepsilon }-\frac{1}{2c^{2}}{\dot{\varepsilon }}^{2}=0.$$

We multiply this equation by *c*^2^ and introduce a new constant $B=S/c^{2}$; then, the dependence of the plastic strain rate on the stress and temperature is described by a nonlinear differential equation:

(114) $$\ddot{\varepsilon }-\frac{1}{B}(\sigma^{ext}+K\theta (T-T_{0}))+\frac{\eta }{B}\dot{\varepsilon }-\frac{1}{2}{\dot{\varepsilon }}^{2}=0.$$

We note that the ratio $\tau_{0}=B/\eta$ determines the relaxation time. In the case of creep with a constant applied force (σ*^ext^* is the specific value of this force) and constant temperature, the quantity $\sigma^{ext}+K\theta (T-T_{0})\equiv a=const$. From the stationary condition, we determine the conditions under which the strain rate is constant. For this, we set $\ddot{\varepsilon }=0$ in Equation (114):

(115) $${\dot{\varepsilon }}^{2}-\frac{2\eta }{B}\dot{\varepsilon }+\frac{2a}{B}=0.$$

From the obtained algebraic equation with respect to the strain rate, we find two solutions:

(116) $${\dot{\varepsilon }}_{1}\equiv p=\frac{\eta }{B}+\sqrt{\frac{\eta_{}^{2}}{B^{2}}-\frac{2\left[\sigma +K\theta \left(T-T_{0}\right)\right]}{B}}\;\mathrm{and}\;{\dot{\varepsilon }}_{2}\equiv q=\frac{\eta }{B}-\sqrt{\frac{\eta^{2}}{B^{2}}-\frac{2\left[\sigma +K\theta \left(T-T_{0}\right)\right]}{B}}.$$

The stationary solution ${\dot{\varepsilon }}_{2}=q$ is stable and ${\dot{\varepsilon }}_{1}=p$ is unstable. The obtained solutions show that there is a critical value of the combination of mechanical and temperature stresses above which creep with a constant rate is impossible:

(117) $${\left[\sigma^{ext}+K\theta (T-T_{0})\right]}^{*}\equiv a^{*}=\frac{\eta^{2}}{2B}.$$

The critical value of the stresses is determined by the material constants, which can be determined experimentally at the stage of steady-state creep. Indeed, determining from experiments two steady-state strain rates $\varsigma_{1}$ and $\varsigma_{2}$ corresponding to two different applied stresses $\sigma_{1}$ and $\sigma_{2}$, we obtain two algebraic equations for *B* and η:

(118) $$\varsigma_{1}{}^{2}-\frac{2\eta }{B}\varsigma_{1}+\frac{2}{B}\sigma_{1}=0\;\mathrm{and}\;\varsigma_{2}{}^{2}-\frac{2\eta }{B}\varsigma_{2}+\frac{2}{B}\sigma_{2}=0,$$

which give an opportunity to get expressions for defining *B* and η:

(119) $$B=2\frac{\left(\sigma_{2}\varsigma_{1}-\sigma_{1}\varsigma_{2}\right)}{\varsigma_{1}\varsigma_{2}(\varsigma_{1}-\varsigma_{2})}\;\mathrm{and}\;\eta =\frac{\sigma_{2}\varsigma_{1}^{2}-\sigma_{1}\varsigma_{2}^{2}}{\varsigma_{1}\varsigma_{2}(\varsigma_{1}-\varsigma_{2})}.$$

To solve Equation (114), it is necessary to know the material constants *B* and η for the considered polymer. It is known from experimental studies that the dependences of creep strain or strain rate on time for different materials are quite similar, which gives an idea of universal equation construction suitable for a large class of materials. It is clear that such equations must be written in dimensionless quantities. We introduce new dimensionless variables $\dot{\xi }=\left(B/\eta \right)\dot{\varepsilon }$, $\tau_{0}=\left(B/\eta \right)$, $\tau =t/\tau_{0}$, and $D=\left(B/\eta^{2}\right)a=a/a^{*}$; thus, Equation (114) with respect to dimensionless variables takes the form

(120) $$\ddot{\xi }=\frac{1}{2}{\dot{\xi }}^{2}-\dot{\xi }+D.$$

The steady-state creep rates are defined as follows:

(121) $${\dot{\xi }}_{1}\equiv p=1+\sqrt{1-2D}\;\mathrm{and}\;{\dot{\xi }}_{2}\equiv q=1-\sqrt{1-2D}.$$

Depending on the ratio of value *a* (determined by mechanical and temperature stresses) to the critical value $a^{*}=\eta^{2}/2B$, we obtain three types of solutions.

The first solution is provided with $a<a^{*}$ or *D* < 0.5. In this case, the solution of Equation (120) under the initial conditions $\xi_{\tau =0}=0$ and ${\dot{\xi }}_{\tau =0}=0$ (zero initial values of strain and strain rate) has the following form (elastic strains are not taken into consideration):

(122) $$\xi =\varepsilon =q\tau -2\ln\left[\frac{p-q\exp\left(-\frac{p-q}{2}\tau \right)}{p-q}\right].$$

**Figure 4:**
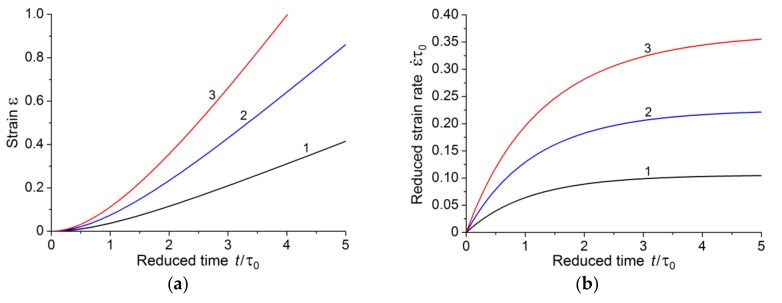
Variations in creep strain ε (**a**) and reduced strain rate $\dot{\xi }=\left(B/\eta \right)\dot{\varepsilon }$ (**b**) with respect to normalized time τ at normalized stresses *D* = 0.1 (1), *D* = 0.2 (2), and *D* = 0.3 (3) lower than the critical value 0.5 ($a<a^{*}$).

For long times, this solution tends to the expression

(123) $$\xi =\varepsilon \approx q\tau -2\ln\left[\frac{p}{p-q}\right],$$

from which it follows that, for larger times, the creep strain linearly depends on time, i.e., the creep mode goes to the steady-state creep stage, and the accelerated creep stage is absent. Figure 4 shows creep curves for three stress values lower than the critical stress.

The second solution, when the condition *D* = 0.5 is satisfied, with the same zero initial strain and strain rate values, has the form

(124) $$\xi =\tau -2\ln\left|1+\frac{\tau }{2}\right|.$$

**Figure 5:**
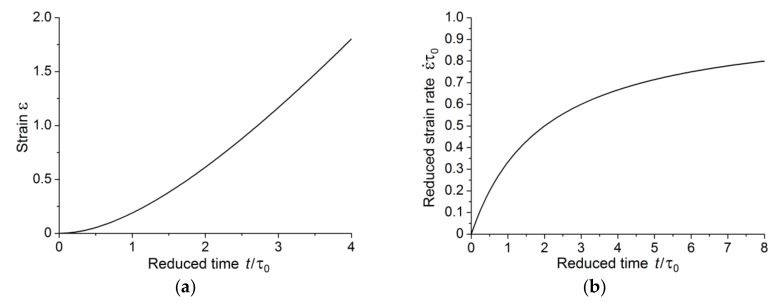
Variations in creep strain ε (**a**) and reduced strain rate $\dot{\xi }=\left(B/\eta \right)\dot{\varepsilon }$ (**b**) with respect to normalized time τ at a critical value of applied stress a = a* (*D* = 0.5).

In this case, the stage of steady creep also takes place and there is no third stage (the accelerated creep stage), as shown in Figure 5.

The third solution takes place at *D* > 0.5 and initial conditions $\xi_{\tau =0}=0$ and ${\dot{\xi }}_{\tau =0}=0$ (zero initial values of strain and strain rate):

(125) $$\xi =\tau -2\ln\left[\cos\frac{\sqrt{2D-1}}{2}\tau +\frac{1}{\sqrt{2D-1}}\sin\frac{\sqrt{2D-1}}{2}\tau \right].$$

**Figure 6:**
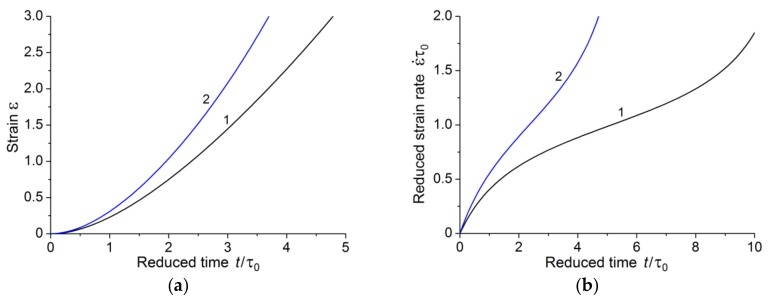
Variations in creep strain ε (**a**) and reduced strain rate $\dot{\xi }=\left(B/\eta \right)\dot{\varepsilon }$ (**b**) with respect to normalized time τ at normalized stresses *D* = 0.6 (1) and *D* = 0.8 (2) above the critical value (a > a*).

Figure 6 shows the dependence of creep strain and strain rate on time at a stress above the critical value.

The above results allow us to make some conclusions. There is a critical stress for each polymer. The value of the critical stress is determined by two material constants; these constants can be determined at the stage of steady creep. If the applied stresses are less than or equal to the critical value, the first stage of creep with subsequent transition to the steady-state creep stage is realized. This is well illustrated in Figure 4b which shows creep strain rate dependence in the case of a < a*. It appears from Figure 4b that creep strain rate tends to the limiting value over time.

If the applied stresses exceed a critical value, then the final stage of creep process is the accelerated creep stage. At stresses moderately exceeding the critical value, we can distinguish three creep stages (curve 1 in Figure 6b). Increase in the value of applied stress far from the critical value is accompanied by the disappearance of the stage of steady-state creep, and the first stage of creep directly passes into the accelerated (tertiary) creep stage (curve 2 in Figure 6b).

The results of the analysis, including the conclusion of critical stress, correspond to the results of experimental studies of the creep of various polymeric materials. Reference [42] describes the results of tensile creep tests on polymer fibers of different composition. The authors particularly discussed the existence of a critical stress (the authors call it “critical creep stress”). They showed that, when approaching it, the length of the steady-state creep stage decreased by orders of magnitude. When the critical value of applied stress was exceeded, this stage disappeared. The authors showed that the magnitude of the critical stress depends on the mechanical (including rheological) properties of the polymer. Similar results were reported for polypropylene samples in References [43,44]. The authors also noted the presence of a threshold stress, above which the steady-state creep stage disappears. The above and other available experimental data confirm the adequacy of the obtained results, including the dependence (Equation (117)) of the value of critical stress on the combination of elastic and inelastic mechanical properties of the polymer.

Note that, to describe the creep process, many theories were proposed, which can be arbitrarily divided into three types: the aging theory, the flow theory, and the hardening theory.

In the aging theory, it is assumed that there is a constant interdependence between the creep strain, the stress, and the time at a certain temperature. In our case, it is shown that such dependences are described by Equations (122), (124) and (125). The form of these dependences essentially depends on the ratio of the applied stresses to the critical stress $a^{*}=\eta^{2}/2B$, which is a combination of material parameters.

In the flow theory, it is assumed that there is a constant interdependence between the creep strain rate, stress, and time. In our case, if the dependences in Equations (122), (124) and (125) are differentiated with respect to time, we obtain the interdependence between the creep strain rate, stress, and time, which is typical of the flow theory.

Therefore, the developed model allows obtaining relationships which are typical of the aging theory and the flow theory.

## 4. Conclusions

Studies carried out in the last decade showed that many of the key mechanical properties of semi-crystalline polymers are determined by the defect structure, in particular, by the concentration, spatial distribution, and defect motion dynamics during the material deformation process. The specific features of the mechanical behavior of semi-crystalline polymers as a two-phase system are determined, among other things, by the specific features of defects in amorphous components and their interaction with defects in crystalline components. This determined the relevance of the development of the approach to the macroscopic description of the inelastic deformation of such materials. This approach takes into account both static (dislocation-type defects) and dynamic (vorticity and acceleration) microscopic mechanisms of the plastic deformation of polymers.

This paper formulated the continuum dynamic model of defects arising during deformation in crystalline and amorphous components of semi-crystalline polymers. Within the framework of the model, the plastic deformation of the crystalline component, realized by means of crystallographic mechanisms, is described by dislocation motion. Unlike most classical models, the present model also takes into account dynamic defects in a viscous amorphous component. The interaction of crystalline and amorphous components determines the sequence of polymer deformation. The first contribution to deformation is given by elastic deformations of the crystalline components and a “defect-free” (laminar type) viscous flow of amorphous components. The process of inelastic polymer deformation is controlled by the motion of dislocation-type defects (in crystalline components) and dynamic defects (in amorphous components).

On the basis of the proposed assumptions, a physical theory of the deformation of semi-crystalline polymers was built. The theory considers the dynamics of defects of various types (static and dynamic) arising in crystalline and amorphous components during material deformation. This theory naturally takes into account the interaction of defects with each other, as well as the interaction of defect fields with stress fields. In particular, we derived expressions for the interaction force of defects with stress fields from external loading. We note that taking into consideration this interaction is one of the key theory advantages since it allows adequately describing polymers with high residual internal stresses. Based on the basic relationships of the theory, we obtained expressions for the energy, impulse, and mass of defects appearing in the crystalline and amorphous components, which indicate that the defect fields are self-dependent objects. Taking into account the inertial characteristics of defects is also an advantage of this theory, and it is especially relevant for an adequate description of polymer mechanical behavior under dynamic loading.

Taking into account the energy, impulse, and mass of the defect fields, a system of interconnected nonlinear equations was obtained, and it describes the macroscopic deformation of a semi-crystalline polymer as a result of the defect dynamics and the interaction between various types of defects. The numerical or analytical solution of this equation system makes it possible to study and predict the material behavior under complex loading conditions, including hardening, softening, and fracture, within the framework of a single (general) formalism, as well as to solve the problems of computer design of the internal structure of composite materials on a polymer basis. Wide capabilities of the developed macroscopic continuum model are illustrated using two simple examples of the analysis of the discrete spectrum of frequencies and energies of natural oscillations of defects in the amorphous phase of the polymer, as well as general features of the creep of polymers with a high degree of crystallinity. The generality of the basic statements and constitutive equations of the developed theory makes it possible to describe the inelastic behavior of polymers over a wide range of crystallinity (from purely amorphous to fully crystalline polymers) under various types of external actions (mechanical or temperature) and different loading regimes (from quasistatic to high strain rate).

## Supplementary Materials

The supplementary materials are available online at http://www.mdpi.com/2073-4360/10/10/1155/s1.
