# Supplementary material for: The Continuum Approach to the Description of Semi-Crystalline Polymers Deformation Regimes: The Role of Dynamic and Translational Defects
Source: Polymers (Basel). 2018 Oct 16;10(10):1155. doi: 10.3390/polym10101155 (PMC6404089; doi:10.3390/polym10101155)
Supplement: Supplementary file 1 [file polymers-10-01155-s001.pdf]

Supplementary Materials for

# The Continuum Approach to the Description of Semi-Crystalline Polymers Deformation Regimes. The Role of Dynamic and Translational Defects

**Yurii V. Grinyaev [1,*], Nadezda V. Chertova [1], Evgeny V. Shilko [1,2] and Sergey G. Psakhie [1]**

[1] Institute of Strength Physics and Materials Science SB RAS, pr. Akademicheskii 2/4. 634055 Tomsk, Russia

[2] National Research Tomsk State University, 36 Lenin ave., 634050 Tomsk, Russia

[*]Corresponding author. E-mail: grn@ispms.tsc.ru

The derivation of many of the mathematical expressions presented in the manuscript is rather cumbersome and is not given in the main body of the paper. In order to make it easier for readers to understand the mathematical procedures for deriving these relations, we give below examples of intermediate algebraic passages to clarify derivations of some equations in the main body of the manuscript.

***1. Derivation of Eq. (38)***

$$\frac{\partial \alpha_{\gamma\tau\nu}}{\partial x^{\mu}} + \frac{\partial \alpha_{\tau\mu\nu}}{\partial x^{\gamma}} + \frac{\partial \alpha_{\mu\gamma\nu}}{\partial x^{\tau}} = 0 . \tag{38}$$

This expression follows from the definition of the defect density tensor (21′). According to this definition:

$$\alpha_{\gamma\tau\nu} = \frac{\partial \beta^{int}_{\tau\nu}}{\partial x^{\gamma}} - \frac{\partial \beta^{int}_{\gamma\nu}}{\partial x^{\tau}} , \qquad \alpha_{\tau\mu\nu} = \frac{\partial \beta^{int}_{\mu\nu}}{\partial x^{\tau}} - \frac{\partial \beta^{int}_{\tau\nu}}{\partial x^{\mu}} , \qquad \alpha_{\mu\tau\nu} = \frac{\partial \beta^{int}_{\tau\nu}}{\partial x^{\mu}} - \frac{\partial \beta^{int}_{\mu\nu}}{\partial x^{\tau}} . \tag{38.1}$$

Summing the corresponding derivatives in Eq. (38.1):

$$\frac{\partial \alpha_{\gamma\tau\nu}}{\partial x^{\mu}} = \frac{\partial}{\partial x^{\mu}}\left(\frac{\partial \beta^{int}_{\tau\nu}}{\partial x^{\gamma}} - \frac{\partial \beta^{int}_{\gamma\nu}}{\partial x^{\tau}}\right) , \quad \frac{\partial \alpha_{\tau\mu\nu}}{\partial x^{\gamma}} = \frac{\partial}{\partial x^{\gamma}}\left(\frac{\partial \beta^{int}_{\mu\nu}}{\partial x^{\tau}} - \frac{\partial \beta^{int}_{\tau\nu}}{\partial x^{\mu}}\right) ,$$
$$\frac{\partial \alpha_{\mu\tau\nu}}{\partial x^{\gamma}} = \frac{\partial}{\partial x^{\gamma}}\left(\frac{\partial \beta^{int}_{\tau\nu}}{\partial x^{\mu}} - \frac{\partial \beta^{int}_{\mu\nu}}{\partial x^{\tau}}\right) , \tag{38.2}$$

we obtain the identity zero, which justifies the correctness of Eq. (38).

***2. Derivation of Eq. (39)***

$$\nabla \times \vec{g} = \frac{\partial \vec{\omega}}{\partial t} \quad \text{and} \quad \nabla \cdot \vec{\omega} = 0 . \tag{39}$$

At ν=0, the identity (38) takes the form

$$\frac{\partial \alpha_{\gamma\tau 0}}{\partial x^{\mu}} + \frac{\partial \alpha_{\tau\mu 0}}{\partial x^{\gamma}} + \frac{\partial \alpha_{\mu\gamma 0}}{\partial x^{\tau}} = 0 . \tag{39.1}$$

At μ=0,1,2,3, the Eq. (39.1) is written as follows:

$$\frac{\partial \alpha_{\gamma\tau 0}}{\partial x^{0}} + \frac{\partial \alpha_{\tau 00}}{\partial x^{\gamma}} + \frac{\partial \alpha_{0\gamma 0}}{\partial x^{\tau}} = 0 , \quad \frac{\partial \alpha_{\gamma\tau 0}}{\partial x^{1}} + \frac{\partial \alpha_{\tau 10}}{\partial x^{\gamma}} + \frac{\partial \alpha_{1\gamma 0}}{\partial x^{\tau}} = 0 ,$$
$$\frac{\partial \alpha_{\gamma\tau 0}}{\partial x^{2}} + \frac{\partial \alpha_{\tau 20}}{\partial x^{\gamma}} + \frac{\partial \alpha_{2\gamma 0}}{\partial x^{\tau}} = 0 , \quad \frac{\partial \alpha_{\gamma\tau 0}}{\partial x^{3}} + \frac{\partial \alpha_{\tau 30}}{\partial x^{\gamma}} + \frac{\partial \alpha_{3\gamma 0}}{\partial x^{\tau}} = 0 . \tag{39.2}$$

At γ=0,1,2,3, the following equalities follows from the first expression in Eq. (39.2):

$$\frac{\partial \alpha_{0\tau 0}}{\partial x^{0}} + \frac{\partial \alpha_{\tau 00}}{\partial x^{0}} + \frac{\partial \alpha_{000}}{\partial x^{\tau}} = 0 , \quad \frac{\partial \alpha_{1\tau 0}}{\partial x^{0}} + \frac{\partial \alpha_{\tau 00}}{\partial x^{1}} + \frac{\partial \alpha_{010}}{\partial x^{\tau}} = 0 ,$$
$$\frac{\partial \alpha_{2\tau 0}}{\partial x^{0}} + \frac{\partial \alpha_{\tau 00}}{\partial x^{2}} + \frac{\partial \alpha_{020}}{\partial x^{\tau}} = 0 , \quad \frac{\partial \alpha_{3\tau 0}}{\partial x^{0}} + \frac{\partial \alpha_{\tau 00}}{\partial x^{3}} + \frac{\partial \alpha_{030}}{\partial x^{\tau}} = 0 . \tag{39.3}$$

Recall that

$$\alpha_{\gamma\tau 0}=\begin{vmatrix}\alpha_{000} & \alpha_{010} & \alpha_{020} & \alpha_{030}\\ \alpha_{100} & \alpha_{110} & \alpha_{120} & \alpha_{130}\\ \alpha_{200} & \alpha_{210} & \alpha_{220} & \alpha_{230}\\ \alpha_{300} & \alpha_{310} & \alpha_{320} & \alpha_{330}\end{vmatrix}=\begin{vmatrix}0 & \frac{1}{c^2}g_1 & \frac{1}{c^2}g_2 & \frac{1}{c^2}g_3\\ -\frac{1}{c^2}g_1 & 0 & \frac{1}{c}\omega_3 & -\frac{1}{c}\omega_2\\ -\frac{1}{c^2}g_2 & -\frac{1}{c}\omega_3 & 0 & \frac{1}{c}\omega_1\\ -\frac{1}{c^2}g_3 & \frac{1}{c}\omega_2 & -\frac{1}{c}\omega_1 & 0\end{vmatrix}. \quad (39.4)$$

At $\tau$=0, all the expressions in (39.3) are identically equal to zero.
At $\tau$=1, they are written as follows:

$$\frac{\partial\alpha_{010}}{\partial x^0}+\frac{\partial\alpha_{100}}{\partial x^0}+\frac{\partial\alpha_{000}}{\partial x^1}=0, \quad \frac{\partial\alpha_{110}}{\partial x^0}+\frac{\partial\alpha_{100}}{\partial x^1}+\frac{\partial\alpha_{010}}{\partial x^1}=0,$$
$$\frac{\partial\alpha_{210}}{\partial x^0}+\frac{\partial\alpha_{100}}{\partial x^2}+\frac{\partial\alpha_{020}}{\partial x^1}=0, \quad \frac{\partial\alpha_{310}}{\partial x^0}+\frac{\partial\alpha_{100}}{\partial x^3}+\frac{\partial\alpha_{030}}{\partial x^1}=0. \quad (39.5)$$

Taking into account Eq. (39.4), Eq. (39.5) can be written in the following form:

$$-\frac{\partial\omega_3}{\partial t}+\frac{\partial g_1}{\partial x^2}-\frac{\partial g_2}{\partial x^1}=0, \quad \frac{\partial\omega_2}{\partial t}-\frac{\partial g_1}{\partial x^3}+\frac{\partial g_3}{\partial x^1}=0. \quad (39.6)$$

Writing Eq. (39.3) in the same way for $\tau$ = 2 and $\tau$ = 3, one can obtain expressions (39.7) and (39.8) respectively:

$$\frac{\partial\omega_3}{\partial t}-\frac{\partial g_2}{\partial x^1}+\frac{\partial g_1}{\partial x^2}=0, \quad -\frac{\partial\omega_1}{\partial t}-\frac{\partial g_2}{\partial x^3}+\frac{\partial g_3}{\partial x^2}=0, \quad (39.7)$$

$$-\frac{\partial\omega_2}{\partial t}-\frac{\partial g_3}{\partial x^1}+\frac{\partial g_1}{\partial x^3}=0, \quad \frac{\partial\omega_1}{\partial t}-\frac{\partial g_3}{\partial x^2}+\frac{\partial g_2}{\partial x^3}=0. \quad (39.8)$$

The expressions (39.6)-(39.8) are componentwise form of the first equation in (39) : $\nabla\times\vec{g}=\frac{\partial\vec{\omega}}{\partial t}$.

At $\gamma$=0,1,2,3, the second equality in Eq. (39.2) implies that:

$$\frac{\partial\alpha_{0\tau 0}}{\partial x^1}+\frac{\partial\alpha_{\tau 10}}{\partial x^0}+\frac{\partial\alpha_{100}}{\partial x^\tau}=0, \quad \frac{\partial\alpha_{1\tau 0}}{\partial x^1}+\frac{\partial\alpha_{\tau 10}}{\partial x^1}+\frac{\partial\alpha_{110}}{\partial x^\tau}=0,$$
$$\frac{\partial\alpha_{2\tau 0}}{\partial x^1}+\frac{\partial\alpha_{\tau 10}}{\partial x^2}+\frac{\partial\alpha_{120}}{\partial x^\tau}=0, \quad \frac{\partial\alpha_{3\tau 0}}{\partial x^1}+\frac{\partial\alpha_{\tau 10}}{\partial x^3}+\frac{\partial\alpha_{130}}{\partial x^\tau}=0. \quad (39.9)$$

At $\tau$=0, 1,2,3, the first equality in (39.9) leads to the following expressions:

$$\frac{\partial\alpha_{000}}{\partial x^1}+\frac{\partial\alpha_{010}}{\partial x^0}+\frac{\partial\alpha_{100}}{\partial x^0}=0, \quad \frac{\partial\alpha_{010}}{\partial x^1}+\frac{\partial\alpha_{110}}{\partial x^0}+\frac{\partial\alpha_{100}}{\partial x^1}=0,$$
$$\frac{\partial\alpha_{020}}{\partial x^1}+\frac{\partial\alpha_{210}}{\partial x^0}+\frac{\partial\alpha_{100}}{\partial x^2}=0, \quad \frac{\partial\alpha_{030}}{\partial x^1}+\frac{\partial\alpha_{310}}{\partial x^0}+\frac{\partial\alpha_{100}}{\partial x^3}=0. \quad (39.10)$$

Taking into account the definition (39.4), we can conclude that the first two expressions are identically equal to zero, and the last two expressions determine the equation obtained earlier:

$$\frac{\partial\omega_2}{\partial t}-\frac{\partial g_1}{\partial x^3}+\frac{\partial g_3}{\partial x^1}=0. \quad (39.11)$$

From the second equality in (39.9) we obtain identically equal to zero equations for $\tau$ = 0,1,2,3:

$$\frac{\partial\alpha_{100}}{\partial x^1}+\frac{\partial\alpha_{010}}{\partial x^1}+\frac{\partial\alpha_{110}}{\partial x^0}=0\,,\quad \frac{\partial\alpha_{110}}{\partial x^1}+\frac{\partial\alpha_{110}}{\partial x^1}+\frac{\partial\alpha_{110}}{\partial x^1}=0\,,$$
$$\frac{\partial\alpha_{120}}{\partial x^1}+\frac{\partial\alpha_{210}}{\partial x^1}+\frac{\partial\alpha_{110}}{\partial x^2}=0\,,\quad \frac{\partial\alpha_{130}}{\partial x^1}+\frac{\partial\alpha_{310}}{\partial x^1}+\frac{\partial\alpha_{110}}{\partial x^3}=0\,. \tag{39.12}$$

From the third equality in (39.9) we obtain identically equal to zero equations for $\tau$ = 0,1,2,3:

$$\frac{\partial\alpha_{200}}{\partial x^1}+\frac{\partial\alpha_{010}}{\partial x^2}+\frac{\partial\alpha_{120}}{\partial x^0}=0\,,\quad \frac{\partial\alpha_{210}}{\partial x^1}+\frac{\partial\alpha_{110}}{\partial x^2}+\frac{\partial\alpha_{120}}{\partial x^1}=0\,,$$
$$\frac{\partial\alpha_{220}}{\partial x^1}+\frac{\partial\alpha_{210}}{\partial x^2}+\frac{\partial\alpha_{120}}{\partial x^2}=0\,,\quad \frac{\partial\alpha_{230}}{\partial x^1}+\frac{\partial\alpha_{310}}{\partial x^2}+\frac{\partial\alpha_{120}}{\partial x^3}=0\,. \tag{39.13}$$

Here the first three equations represent identically zero equalities, and the last one defines the expression:

$$\frac{\partial\omega_1}{\partial x^1}+\frac{\partial\omega_2}{\partial x^2}+\frac{\partial\omega_3}{\partial x^3}=0\,, \tag{39.14}$$

or in vector form:

$$\nabla\cdot\vec{\omega}=0\,.$$

This corresponds to the second equation in (39) статьи.
Note that the last equation in (39.9) is followed by expressions:

$$\frac{\partial\alpha_{300}}{\partial x^1}+\frac{\partial\alpha_{010}}{\partial x^3}+\frac{\partial\alpha_{130}}{\partial x^0}=0\,,\quad \frac{\partial\alpha_{310}}{\partial x^1}+\frac{\partial\alpha_{110}}{\partial x^3}+\frac{\partial\alpha_{130}}{\partial x^1}=0\,,$$
$$\frac{\partial\alpha_{320}}{\partial x^1}+\frac{\partial\alpha_{210}}{\partial x^3}+\frac{\partial\alpha_{130}}{\partial x^2}=0\,,\quad \frac{\partial\alpha_{330}}{\partial x^1}+\frac{\partial\alpha_{310}}{\partial x^3}+\frac{\partial\alpha_{130}}{\partial x^3}=0\,, \tag{39.15}$$

which determine the equations (39.11) and (39.14).

### *3. Derivation of Eq. (40)*

$$\nabla\cdot\tilde{\alpha}=0 \quad\text{and}\quad \nabla\times J=\frac{\partial\tilde{\alpha}}{\partial t}\,. \tag{40}$$

Let us consider Eq. (38). We will consider the spatial values of the index $\nu=i$=1,2,3 (here the indices $\gamma,\mu,\tau$ take the values 0,1,2,3).
At $\gamma$=0, the possible variants of the formula (38) are following equalities:
a) at $\mu$=0 and $\tau$=0,1,2,3

$$\frac{\partial\alpha_{00i}}{\partial x^0}+\frac{\partial\alpha_{00i}}{\partial x^0}+\frac{\partial\alpha_{00i}}{\partial x^0}=0\,,\quad \frac{\partial\alpha_{01i}}{\partial x^1}+\frac{\partial\alpha_{11i}}{\partial x^0}+\frac{\partial\alpha_{10i}}{\partial x^1}=0\,,$$
$$\frac{\partial\alpha_{02i}}{\partial x^2}+\frac{\partial\alpha_{22i}}{\partial x^0}+\frac{\partial\alpha_{20i}}{\partial x^2}=0\,,\quad \frac{\partial\alpha_{03i}}{\partial x^3}+\frac{\partial\alpha_{33i}}{\partial x^0}+\frac{\partial\alpha_{30i}}{\partial x^3}=0\,; \tag{40.1}$$

b) at $\mu$=1 and $\tau$=0,1,2,3

$$\frac{\partial\alpha_{00i}}{\partial x^1}+\frac{\partial\alpha_{01i}}{\partial x^0}+\frac{\partial\alpha_{10i}}{\partial x^0}=0\,,\quad \frac{\partial\alpha_{01i}}{\partial x^1}+\frac{\partial\alpha_{11i}}{\partial x^0}+\frac{\partial\alpha_{10i}}{\partial x^1}=0\,,$$
$$\frac{\partial\alpha_{02i}}{\partial x^1}+\frac{\partial\alpha_{21i}}{\partial x^0}+\frac{\partial\alpha_{10i}}{\partial x^2}=0\,,\quad \frac{\partial\alpha_{03i}}{\partial x^1}+\frac{\partial\alpha_{31i}}{\partial x^0}+\frac{\partial\alpha_{10i}}{\partial x^3}=0\,; \tag{40.2}$$

c) at $\mu$=2 and $\tau$=0,1,2,3

$$\frac{\partial\alpha_{00i}}{\partial x^2}+\frac{\partial\alpha_{02i}}{\partial x^0}+\frac{\partial\alpha_{20i}}{\partial x^0}=0\,,\quad \frac{\partial\alpha_{01i}}{\partial x^0}+\frac{\partial\alpha_{10i}}{\partial x^0}+\frac{\partial\alpha_{00i}}{\partial x^1}=0\,,$$
$$\frac{\partial\alpha_{02i}}{\partial x^0}+\frac{\partial\alpha_{20i}}{\partial x^0}+\frac{\partial\alpha_{00i}}{\partial x^2}=0\,,\quad \frac{\partial\alpha_{03i}}{\partial x^0}+\frac{\partial\alpha_{30i}}{\partial x^0}+\frac{\partial\alpha_{00i}}{\partial x^3}=0\,; \tag{40.3}$$

d) at μ=3 and τ=0,1,2,3

$$\frac{\partial\alpha_{00i}}{\partial x^3}+\frac{\partial\alpha_{03i}}{\partial x^0}+\frac{\partial\alpha_{30i}}{\partial x^0}=0\,,\quad \frac{\partial\alpha_{01i}}{\partial x^3}+\frac{\partial\alpha_{13i}}{\partial x^0}+\frac{\partial\alpha_{30i}}{\partial x^1}=0\,,$$
$$\frac{\partial\alpha_{02i}}{\partial x^3}+\frac{\partial\alpha_{23i}}{\partial x^0}+\frac{\partial\alpha_{30i}}{\partial x^2}=0\,,\quad \frac{\partial\alpha_{03i}}{\partial x^3}+\frac{\partial\alpha_{33i}}{\partial x^0}+\frac{\partial\alpha_{30i}}{\partial x^3}=0\,. \tag{40.4}$$

Recall that

$$\alpha_{\gamma\tau i}=\begin{vmatrix}\alpha_{00i} & \alpha_{01i} & \alpha_{02i} & \alpha_{03i}\\ \alpha_{10i} & \alpha_{11i} & \alpha_{12i} & \alpha_{13i}\\ \alpha_{20i} & \alpha_{21i} & \alpha_{22i} & \alpha_{23i}\\ \alpha_{30i} & \alpha_{31i} & \alpha_{32i} & \alpha_{33i}\end{vmatrix}=\begin{vmatrix}0 & \frac{1}{c}J_{1i} & \frac{1}{c}J_{2i} & \frac{1}{c}J_{3i}\\ -\frac{1}{c}J_{1i} & 0 & \tilde{\alpha}_{3i} & -\tilde{\alpha}_{2i}\\ -\frac{1}{c}J_{2i} & -\tilde{\alpha}_{3i} & 0 & \tilde{\alpha}_{1i}\\ -\frac{1}{c}J_{3i} & \tilde{\alpha}_{2i} & -\tilde{\alpha}_{1i} & 0\end{vmatrix}. \tag{40.5}$$

Substituting the values of the elements of the matrix (40.5) in (40.1)-(40.4), we obtain the following equations:

$$\frac{\partial J_{2i}}{\partial x^1}-\frac{\partial\tilde{\alpha}_{3i}}{\partial x^0}-\frac{\partial J_{1i}}{\partial x^2}=0\,,\quad \frac{\partial J_{3i}}{\partial x^1}+\frac{\partial\tilde{\alpha}_{2i}}{\partial x^0}-\frac{\partial J_{1i}}{\partial x^3}=0\,,\quad \frac{\partial J_{2i}}{\partial x^3}+\frac{\partial\alpha_{1i}}{\partial t}-\frac{\partial J_{3i}}{\partial x^2}=0\,, \tag{40.6}$$

which correspond to the second vector equality in Eq. (40).
At γ=1, the possible variants of the formula (38) are following equalities:
a) at μ=0 and τ=0,1,2,3

$$\frac{\partial\alpha_{10i}}{\partial x^0}+\frac{\partial\alpha_{00i}}{\partial x^1}+\frac{\partial\alpha_{01i}}{\partial x^0}=0\,,\quad \frac{\partial\alpha_{11i}}{\partial x^0}+\frac{\partial\alpha_{10i}}{\partial x^1}+\frac{\partial\alpha_{01i}}{\partial x^1}=0\,,$$
$$\frac{\partial\alpha_{12i}}{\partial x^0}+\frac{\partial\alpha_{20i}}{\partial x^1}+\frac{\partial\alpha_{01i}}{\partial x^2}=0\,,\quad \frac{\partial\alpha_{13i}}{\partial x^0}+\frac{\partial\alpha_{30i}}{\partial x^1}+\frac{\partial\alpha_{01i}}{\partial x^3}=0\,; \tag{40.7}$$

b) at μ=1 and τ=0,1,2,3

$$\frac{\partial\alpha_{10i}}{\partial x^1}+\frac{\partial\alpha_{01i}}{\partial x^1}+\frac{\partial\alpha_{11i}}{\partial x^0}=0\,,\quad \frac{\partial\alpha_{11i}}{\partial x^1}+\frac{\partial\alpha_{11i}}{\partial x^1}+\frac{\partial\alpha_{11i}}{\partial x^1}=0\,,$$
$$\frac{\partial\alpha_{12i}}{\partial x^1}+\frac{\partial\alpha_{21i}}{\partial x^1}+\frac{\partial\alpha_{11i}}{\partial x^2}=0\,,\quad \frac{\partial\alpha_{13i}}{\partial x^1}+\frac{\partial\alpha_{31i}}{\partial x^1}+\frac{\partial\alpha_{11i}}{\partial x^3}=0\,; \tag{40.8}$$

c) at μ=2 and τ=0,1,2,3

$$\frac{\partial\alpha_{10i}}{\partial x^2}+\frac{\partial\alpha_{02i}}{\partial x^1}+\frac{\partial\alpha_{21i}}{\partial x^0}=0\,,\quad \frac{\partial\alpha_{11i}}{\partial x^2}+\frac{\partial\alpha_{12i}}{\partial x^1}+\frac{\partial\alpha_{21i}}{\partial x^1}=0\,,$$
$$\frac{\partial\alpha_{12i}}{\partial x^2}+\frac{\partial\alpha_{22i}}{\partial x^1}+\frac{\partial\alpha_{21i}}{\partial x^2}=0\,,\quad \frac{\partial\alpha_{13i}}{\partial x^2}+\frac{\partial\alpha_{32i}}{\partial x^1}+\frac{\partial\alpha_{21i}}{\partial x^3}=0\,; \tag{40.9}$$

d) at μ=3 and τ=0,1,2,3

$$\frac{\partial\alpha_{10i}}{\partial x^3}+\frac{\partial\alpha_{03i}}{\partial x^1}+\frac{\partial\alpha_{31i}}{\partial x^0}=0\,,\quad \frac{\partial\alpha_{11i}}{\partial x^3}+\frac{\partial\alpha_{13i}}{\partial x^1}+\frac{\partial\alpha_{31i}}{\partial x^1}=0\,, \tag{40.10}$$

$$\frac{\partial\alpha_{12i}}{\partial x^3}+\frac{\partial\alpha_{23i}}{\partial x^1}+\frac{\partial\alpha_{31i}}{\partial x^2}=0 \;\cdot\; , \frac{\partial\alpha_{13i}}{\partial x^3}+\frac{\partial\alpha_{33i}}{\partial x^1}+\frac{\partial\alpha_{31i}}{\partial x^3}=0$$

Substituting the corresponding elements of the matrix (40.5) in (40.7)-(40.10), we obtain, along with the zero identities and the previously obtained equalities

$$\frac{\partial\tilde\alpha_{3i}}{\partial t}-\frac{\partial J_{2i}}{\partial x^1}+\frac{\partial J_{1i}}{\partial x^2}=0\;,\; -\frac{\partial\tilde\alpha_{2i}}{\partial x^0}-\frac{\partial J_{3i}}{\partial x^1}+\frac{\partial J_{1i}}{\partial x^3}=0\;,$$

the equation:

$$\frac{\partial\tilde\alpha_{3i}}{\partial x^3}+\frac{\partial\tilde\alpha_{1i}}{\partial x^1}+\frac{\partial\tilde\alpha_{2i}}{\partial x^2}=0 \quad\text{or}\quad \nabla\cdot\tilde\alpha=0. \tag{40.11}$$

This equation is the first expression in (40).
Below we show that the same result is obtained for other values of $\gamma$ ($\gamma$=2,3).
Indeed, if $\gamma$=2, then:
a) at $\mu$=0 and $\tau$=0,1,2,3, from (38) we have the equalities

$$\frac{\partial\alpha_{20i}}{\partial x^0}+\frac{\partial\alpha_{00i}}{\partial x^2}+\frac{\partial\alpha_{02i}}{\partial x^0}=0\;,\; \frac{\partial\alpha_{21i}}{\partial x^0}+\frac{\partial\alpha_{10i}}{\partial x^2}+\frac{\partial\alpha_{02i}}{\partial x^1}=0\;,$$
$$\frac{\partial\alpha_{22i}}{\partial x^0}+\frac{\partial\alpha_{20i}}{\partial x^2}+\frac{\partial\alpha_{02i}}{\partial x^2}=0\;,\; \frac{\partial\alpha_{23i}}{\partial x^0}+\frac{\partial\alpha_{30i}}{\partial x^2}+\frac{\partial\alpha_{02i}}{\partial x^3}=0\,; \tag{40.12}$$

b) at $\mu$=1 and $\tau$=0,1,2,3

$$\frac{\partial\alpha_{20i}}{\partial x^1}+\frac{\partial\alpha_{01i}}{\partial x^2}+\frac{\partial\alpha_{12i}}{\partial x^0}=0\;,\; \frac{\partial\alpha_{21i}}{\partial x^1}+\frac{\partial\alpha_{11i}}{\partial x^2}+\frac{\partial\alpha_{12i}}{\partial x^1}=0\;,$$
$$\frac{\partial\alpha_{22i}}{\partial x^1}+\frac{\partial\alpha_{21i}}{\partial x^2}+\frac{\partial\alpha_{12i}}{\partial x^2}=0\;,\; \frac{\partial\alpha_{23i}}{\partial x^1}+\frac{\partial\alpha_{31i}}{\partial x^2}+\frac{\partial\alpha_{12i}}{\partial x^3}=0\,; \tag{40.13}$$

c) at $\mu$=2 and $\tau$=0,1,2,3

$$\frac{\partial\alpha_{20i}}{\partial x^2}+\frac{\partial\alpha_{02i}}{\partial x^2}+\frac{\partial\alpha_{22i}}{\partial x^0}=0\;,\; \frac{\partial\alpha_{21i}}{\partial x^2}+\frac{\partial\alpha_{12i}}{\partial x^2}+\frac{\partial\alpha_{22i}}{\partial x^1}=0\;,$$
$$\frac{\partial\alpha_{22i}}{\partial x^2}+\frac{\partial\alpha_{22i}}{\partial x^2}+\frac{\partial\alpha_{22i}}{\partial x^2}=0\;,\; \frac{\partial\alpha_{23i}}{\partial x^2}+\frac{\partial\alpha_{32i}}{\partial x^2}+\frac{\partial\alpha_{22i}}{\partial x^3}=0\,; \tag{40.14}$$

d) at $\mu$=3 and $\tau$=0,1,2,3

$$\frac{\partial\alpha_{20i}}{\partial x^3}+\frac{\partial\alpha_{03i}}{\partial x^2}+\frac{\partial\alpha_{32i}}{\partial x^0}=0\;,\; \frac{\partial\alpha_{21i}}{\partial x^3}+\frac{\partial\alpha_{13i}}{\partial x^2}+\frac{\partial\alpha_{32i}}{\partial x^1}=0\;,$$
$$\frac{\partial\alpha_{22i}}{\partial x^3}+\frac{\partial\alpha_{23i}}{\partial x^2}+\frac{\partial\alpha_{32i}}{\partial x^2}=0\;,\; \frac{\partial\alpha_{23i}}{\partial x^3}+\frac{\partial\alpha_{33i}}{\partial x^2}+\frac{\partial\alpha_{32i}}{\partial x^3}=0\,. \tag{40.15}$$

When we substitute the values of the elements of the matrix (40.5) into (40.12)-(40.15), we obtain the already known equations:

$$\frac{\partial\tilde\alpha_{3i}}{\partial t}+\frac{\partial J_{1i}}{\partial x^2}-\frac{\partial J_{2i}}{\partial x^1}=0\;,\; \frac{\partial\tilde\alpha_{1i}}{\partial t}-\frac{\partial J_{3i}}{\partial x^2}+\frac{\partial J_{2i}}{\partial x^3}=0\;,$$
$$\frac{\partial\alpha_{2i}}{\partial x^3}-\frac{\partial J_{3i}}{\partial x^2}+\frac{\partial\tilde\alpha_{1i}}{\partial t}=0\;,\; \frac{\partial\tilde\alpha_{1i}}{\partial x^1}+\frac{\partial\tilde\alpha_{2i}}{\partial x^2}+\frac{\partial\tilde\alpha_{3i}}{\partial x^3}=0\,. \tag{40.16}$$

Writing (38) in the same way for $\gamma$=3 and $\mu,\tau$=0,1,2,3, we also obtain the previously found equalities:

$$\frac{\partial\tilde\alpha_{2i}}{\partial t}-\frac{\partial J_{1i}}{\partial x^3}+\frac{\partial J_{3i}}{\partial x^1}=0\;,\; \frac{\partial\tilde\alpha_{1i}}{\partial t}-\frac{\partial J_{2i}}{\partial x^3}+\frac{\partial J_{3i}}{\partial x^2}=0\;,\; \frac{\partial\tilde\alpha_{1i}}{\partial x^1}+\frac{\partial\tilde\alpha_{2i}}{\partial x^2}+\frac{\partial\tilde\alpha_{3i}}{\partial x^3}=0\,. \tag{40.17}$$

*4. Derivation of Eq. (42)*

$$\frac{\partial \sigma_{\tau\nu}}{\partial x_\tau} = 0 . \tag{42}$$

Formula (42) follows from Eq. (41) and the definition of the defect density tensor (38.1):

$$\alpha_{\gamma\tau\nu} = \frac{\partial \beta_{\tau\nu}}{\partial x^\gamma} - \frac{\partial \beta_{\gamma\nu}}{\partial x^\tau}, \quad \frac{\partial \alpha_{\gamma\tau\nu}}{\partial x^\gamma} = \frac{\partial}{\partial x^\gamma}\Big(\frac{\partial \beta_{\tau\nu}}{\partial x^\gamma} - \frac{\partial \beta_{\gamma\nu}}{\partial x^\tau}\Big). \tag{42.1}$$

Taking these expressions into account, the Eq. (41) can be written in the form:

$$\frac{\partial \alpha_{\gamma\tau\nu}}{\partial x^\gamma} = \frac{\partial}{\partial x^\gamma}\Big(\frac{\partial \beta_{\tau\nu}}{\partial x^\gamma} - \frac{\partial \beta_{\gamma\nu}}{\partial x^\tau}\Big) = \frac{1}{S}\sigma_{\tau\nu} . \tag{42.2}$$

Determining the divergence of this expression, we get the relation:

$$\frac{1}{S}\frac{\partial \sigma_{\tau\nu}}{\partial x^\tau} = \frac{\partial^2}{\partial x^\gamma \partial x^\tau}\Big(\frac{\partial \beta_{\tau\nu}}{\partial x^\gamma} - \frac{\partial \beta_{\gamma\nu}}{\partial x^\tau}\Big) = \frac{\partial^2 \partial \beta_{\tau\nu}}{\partial^2 x^\gamma \partial x^\tau} - \frac{\partial^2 \beta_{\gamma\nu}}{\partial^2 x^\tau \partial x^\gamma} \equiv 0 , \tag{42.3}$$

which corresponds to the equation (42).

*5. Derivation of Eq. (51)*

$$\nabla \cdot \vec{g} = \frac{c^4}{S}\rho . \tag{51}$$

This formula is derived as follows. At $\tau,\nu=0$, from (42.2) we get:

$$\frac{\partial \alpha_{000}}{\partial x^0} + \frac{\partial \alpha_{100}}{\partial x^1} + \frac{\partial \alpha_{200}}{\partial x^2} + \frac{\partial \alpha_{300}}{\partial x^3} = \frac{1}{S}\sigma_{00} . \tag{51.1}$$

Substituting here the corresponding elements of the matrix (39.4) and the value of the corresponding component of the stress tensor (see Eq. (48) in the manuscript)

$$\sigma_{\tau\nu} = \begin{vmatrix} \sigma_{00} & \sigma_{01} & \sigma_{02} & \sigma_{03} \\ \sigma_{10} & \sigma_{11} & \sigma_{12} & \sigma_{13} \\ \sigma_{20} & \sigma_{21} & \sigma_{22} & \sigma_{23} \\ \sigma_{30} & \sigma_{31} & \sigma_{32} & \sigma_{33} \end{vmatrix} = \begin{vmatrix} -c^2\rho & c\rho V_1^* & c\rho V_2^* & c\rho V_3^* \\ c\rho V_1^* & \sigma_{11} & \sigma_{12} & \sigma_{13} \\ c\rho V_2^* & \sigma_{21} & \sigma_{22} & \sigma_{23} \\ c\rho V_3^* & \sigma_{31} & \sigma_{32} & \sigma_{33} \end{vmatrix} , \tag{51.2}$$

we get:

$$\frac{1}{c^2}\Big(\frac{\partial g_1}{\partial x^1} + \frac{\partial g_2}{\partial x^2} + \frac{\partial g_3}{\partial x^3}\Big) = \frac{c^2}{S}\rho . \tag{51.3}$$

Vector form of this expression coincides with (51).

*6. Derivation of Eq. (54)*

$$\nabla \times \vec{\omega} = -\frac{1}{c^2}\frac{\partial \vec{g}}{\partial t} - \frac{c^2}{S}\rho \vec{V}^* . \tag{54}$$

This formula is derived similar to (51).
May $\tau$=i take spatial indices (e.g. $i$=1) then we get on the basis on (42.2):

$$\frac{\partial \alpha_{010}}{\partial x^0}+\frac{\partial \alpha_{110}}{\partial x^1}+\frac{\partial \alpha_{210}}{\partial x^2}+\frac{\partial \alpha_{310}}{\partial x^3}=\frac{1}{S}\sigma_{10}. \quad (54.1)$$

Substituting the values of the elements of the matrix (39.4), we obtain the following expression:

$$-\frac{1}{c^3}\frac{\partial g_1}{\partial t}-\frac{1}{c}\frac{\partial \omega_3}{\partial x^2}+\frac{1}{c}\frac{\partial \omega_2}{\partial x^3}=\frac{1}{S}\sigma_{10}. \quad (54.2)$$

For other values $i$=2,3, we get similar expressions

$$\frac{\partial \alpha_{020}}{\partial x^0}+\frac{\partial \alpha_{120}}{\partial x^1}+\frac{\partial \alpha_{220}}{\partial x^2}+\frac{\partial \alpha_{320}}{\partial x^3}=\frac{1}{S}\sigma_{20}, \quad \frac{\partial \alpha_{030}}{\partial x^0}+\frac{\partial \alpha_{130}}{\partial x^1}+\frac{\partial \alpha_{230}}{\partial x^2}+\frac{\partial \alpha_{330}}{\partial x^3}=\frac{1}{S}\sigma_{30},$$

which lead to

$$-\frac{1}{c^3}\frac{\partial g_2}{\partial t}+\frac{1}{c}\frac{\partial \omega_3}{\partial x^1}-\frac{1}{c}\frac{\partial \omega_1}{\partial x^3}=\frac{1}{S}\sigma_{20}, \quad -\frac{1}{c^3}\frac{\partial g_3}{\partial t}-\frac{1}{c}\frac{\partial \omega_2}{\partial x^1}+\frac{1}{c}\frac{\partial \omega_1}{\partial x^2}=\frac{1}{S}\sigma_{30}. \quad (54.3)$$

Together with (54.2) and taking into account the definitions of the components $\sigma_{i0}$ (see Eq. (51.2)), we obtain the expression (54).

*7. Derivation of Eq. (55)*

$$\nabla\cdot J=-\frac{c^2}{S}\rho\vec{V}^*. \quad (55)$$

If we assume that the index ν takes spatial values and τ=0 in (41) (or (42.2) in Supplementary Materials), we can write the following expression

$$\frac{\partial \alpha_{\gamma 0\nu}}{\partial x^\gamma}=\frac{\partial \alpha_{00\nu}}{\partial x^0}+\frac{\partial \alpha_{10\nu}}{\partial x^1}+\frac{\partial \alpha_{20\nu}}{\partial x^2}+\frac{\partial \alpha_{30\nu}}{\partial x^3}=\frac{1}{S}\sigma_{0\nu}. \quad (55.1)$$

Substituting the corresponding values from (40.5) and (51.2) for $\alpha_{0\nu\gamma}$ and $\sigma_{0\nu}$ respectively, we obtain an equation for the tensor of translational defects flux density in crystalline phase:

$$\frac{\partial J_{1\nu}}{\partial x^1}+\frac{\partial J_{2\nu}}{\partial x^2}+\frac{\partial J_{3\nu}}{\partial x^3}=-\frac{c^2}{S}\rho V_\nu^{\;*}, \quad \text{or} \quad \frac{\partial J_{1\nu}}{\partial x^1}+\frac{\partial J_{2\nu}}{\partial x^2}+\frac{\partial J_{3\nu}}{\partial x^3}=-\frac{c^2}{S}\rho V_\nu^{\;*}. \quad (55.2)$$

*8. Derivation of Eq. (56)*

$$\nabla\times\tilde{\alpha}=-\frac{1}{c^2}\frac{\partial J}{\partial t}-\frac{1}{S}\sigma. \quad (56)$$

This formula is obtained similarly to (55) when the indices ν and τ in equation (51.2) take only spatial values:

$$\frac{\partial \alpha_{\gamma ij}}{\partial x^\gamma}=\frac{\partial \alpha_{0ij}}{\partial x^0}+\frac{\partial \alpha_{1ij}}{\partial x^1}+\frac{\partial \alpha_{2ij}}{\partial x^2}+\frac{\partial \alpha_{3ij}}{\partial x^3}=\frac{1}{S}\sigma_{ij}, \quad (56.1)$$

where $i,j$=1,2,3.
Replacing $\alpha_{\gamma ij}$ with the corresponding values from the table (40.5), we obtain the required expression (56).

***8. Derivation of Eq. (59)***

$$F^{\nu} = \sigma^{\tau 0}\alpha^{\nu}_{\tau 0} . \tag{59}$$

This equation is written for the time component of the expression (58). Note that Eq. (58) is a four-dimensional generalization of the well-known expression for three-dimensional space. The continuous three-dimensional expression for the force acting on the ensemble of defects can be obtained on the basis of the classical expression for the force acting on a single defect (see the paper M. Peach and J. S. Koehler The Forces Exerted on Dislocations and the Stress Fields Produced by Them // *Phys. Rev.* **1950**, *80* ,436).

***9. Derivation of Eq. (60)***

$$F^{0} = \frac{\rho}{c}\vec{V}^{*}\cdot\vec{g} . \tag{60}$$

This equation follows from Eq. (59) at ν=0:

$$F^{0} = \sigma^{\tau 0}\alpha^{0}_{\tau 0} = \sigma^{00}\alpha^{0}_{00} + \sigma^{10}\alpha^{0}_{10} + \sigma^{20}\alpha^{0}_{20} + +\sigma^{30}\alpha^{0}_{30} , \tag{60.1}$$

when substituting corresponding values of the components of the matrices $\sigma^{\tau 0}$ and $\alpha^{0}{}_{\tau 0}$ (see Eqs. (39.4) and (51.2)).

***10. Derivation of Eq. (63)***

$$\frac{\partial}{\partial t}\frac{S}{2}\left(\frac{1}{c^{4}}g^{2} + \frac{1}{c^{2}}\omega^{2}\right) = -\nabla\cdot\left(\frac{S}{c^{2}}\vec{\omega}\times\vec{g}\right) - \rho\vec{V}^{*}\cdot\vec{g} . \tag{63}$$

The sequence of steps to obtain this formula is described in the main body of the paper.

***11. Derivation of Eq. (66)***

$$\vec{F} = \rho\vec{g} + \vec{\omega}\times\rho\vec{V}^{*} . \tag{66}$$

This formula follows from Eq. (59) at spatial values of the index ν=*i*:

$$F^{i} = \sigma^{\tau 0}\alpha^{i}_{\tau 0} = \sigma^{00}\alpha^{i}_{00} + \sigma^{10}\alpha^{i}_{10} + \sigma^{20}\alpha^{i}_{20} + +\sigma^{30}\alpha^{i}_{30} , \tag{66.1}$$

when substituting corresponding values of the components of the matrices $\sigma^{\tau 0}$ and $\alpha^{i}{}_{\tau 0}$ (see Eqs. (40.5) and (51.2)).

***12. Derivation of Eq. (80)***

$$\frac{\partial}{\partial t}\frac{S}{2}\left(\frac{1}{c^{2}}J^{2} + \alpha^{2}\right) = \sigma\cdot\cdot\frac{\partial\beta^{pl}}{\partial t} - \nabla\cdot S(\alpha\times\cdot J) . \tag{80}$$

Formula (80) is derived from (76), which is a partial case of Eq. (59) at the spatial value of γ and ν=0. The procedure of obtaining this expression in the work is typical.
Recall that the mathematical symbol "··" denotes the double scalar convolution of tensors, and the

symbol "×·" denotes the vector product of tensors with respect to the first indices and the scalar product with respect to the second indices.

***13. Derivation of Eq. (84)***

$$\vec{F} = (\sigma \times \cdot \tilde{\alpha}) + \rho J \cdot \vec{V}^* . \tag{84}$$

This expression is derived on the basis of Eq. (76) at spatial values of the index *γ=j*:

$$F^j = \sigma^{\tau i}\alpha^j_{\tau i} = \sigma^{01}\alpha^j_{01} + \sigma^{02}\alpha^j_{02} + \sigma^{03}\alpha^j_{03} + \sigma^{11}\alpha^j_{11} + \sigma^{12}\alpha^j_{12} + \sigma^{13}\alpha^j_{13} + ...\sigma^{31}\alpha^j_{31} + \sigma^{02}\alpha^j_{02} + \sigma^{33}\alpha^j_{33} . \tag{84.1}$$

Taking into account the definitions of the corresponding components of the matrices, Eq. (84.1) transforms to (84).

***14. Derivation of Eq. (88)***

$$\vec{F} = -S\nabla \cdot \left( \frac{1}{c^2} J \cdot J + \tilde{\alpha} \cdot \tilde{\alpha} \right) + \frac{S}{2}\left( \frac{1}{c^2}\nabla J^2 + \nabla \tilde{\alpha}^2 \right) + \frac{S}{c^2}\frac{\partial}{\partial t}\tilde{\alpha} \times \cdot J . \tag{88}$$

Derivation of this formula is typical and not difficult with the above explanations on the mathematical symbols "··" и "×·".

***15-16. Derivation of Eqs. (95)-(96)***

$$\frac{\partial}{\partial t}(\rho + n_1\rho_1 + n_2\rho_2) + \nabla \cdot (\rho \vec{V}^* + n_2\vec{p}^{(2)} + n_1\vec{p}^{(1)}) = 0 . \tag{95}$$

$$\nabla \cdot \left[\sigma + \sigma^{(1)} + \sigma^{(2)}\right] = \frac{\partial}{\partial t}\left[\rho \vec{V}^* + n_2\vec{p}^{(2)} + n_1\vec{p}^{(1)}\right] . \tag{96}$$

These equations are derived in the similar way.
In particular, to obtain (95) we take the divergence operation from equation (93c):

$$\nabla \cdot (\nabla \times \vec{\omega}) = \nabla \cdot \left( -\frac{1}{c^2}\frac{\partial \vec{g}}{\partial t} - \frac{c^2}{S}\rho \vec{V}^* - n_2\frac{c^2}{S}\vec{p}^{(2)} - n_1\frac{c^2}{S}\vec{p}^{(1)} \right) . \tag{95.1}$$

The left side of this equation is identically zero: $\nabla \cdot (\nabla \times \vec{\omega}) \equiv 0$. In the right side, we swap the derivatives with respect to coordinates and time:

$$0 = \frac{1}{c^2}\frac{\partial(\nabla \cdot \vec{g})}{\partial t}\nabla \cdot \left( \frac{c^2}{S}\rho \vec{V}^* + n_2\frac{c^2}{S}\vec{p}^{(2)} + n_1\frac{c^2}{S}\vec{p}^{(1)} \right) . \tag{95.2}$$

Then we use the definition of $(\nabla \cdot \vec{g})$ from equation (93d) and obtain the desired equation (95).
To obtain (96) we take the divergence operation from equation (93h):

$$\nabla \cdot (\nabla \times \tilde{\alpha}) = \nabla \cdot \left( -\frac{1}{c^2}\frac{\partial J}{\partial t} - \frac{1}{S}\sigma - \frac{1}{S}\sigma^{(1)} - \frac{1}{S}\sigma^{(2)} \right) . \tag{96.1}$$

The left side of this equation is identically zero: $\nabla \cdot (\nabla \times \tilde{\alpha}) \equiv 0$. In the right side, we swap the derivatives with respect to coordinates and time:

$$0 = \frac{1}{c^2}\frac{\partial(\nabla \cdot J)}{\partial t} + \nabla \cdot \left( \frac{1}{S}\sigma + \frac{1}{S}\sigma^{(1)} + \frac{1}{S}\sigma^{(2)} \right) . \tag{96.2}$$

Then we use the definition of $(\nabla \cdot J)$ from equation (93g) and obtain the desired equation (96).

***17. Derivation of Eq. (111)***

$$\frac{1}{c^2}\frac{\partial J_{xx}}{\partial t}+\frac{1}{S}\sigma_{xx}+\frac{1}{2c^2}J_{xx}^2=0\,. \tag{111}$$

In the revised version of the paper, Eq. (111) is written for the particular component of the tensor ($J_{xx}$) under condition of uniaxial loading.

***18. Derivation of Eq. (120)***

$$\ddot{\xi}=\frac{1}{2}\dot{\xi}^2-\dot{\xi}+D\,. \tag{120}$$

This equation is a formulation of Eq. (114) after the introduced new designations and taking into account the temperature and viscous stresses.
